# Ninjurin1 Plays a Crucial Role in Pulmonary Fibrosis by Promoting Interaction between Macrophages and Alveolar Epithelial Cells

**DOI:** 10.1038/s41598-018-35997-x

**Published:** 2018-12-03

**Authors:** Seungho Choi, Jong Kyu Woo, Yeong-Su Jang, Ju-Hee Kang, Jong-Ik Hwang, Je Kyung Seong, Yeo Sung Yoon, Seung Hyun Oh

**Affiliations:** 1 College of Veterinary Medicine, Seoul National University, Seoul, Republic of Korea; 2 Korea Mouse Phenotyping Center, College of Veterinary Medicine, Seoul National University, Seoul, Republic of Korea; 3 College of Pharmacy, Gachon University, Incheon, Republic of Korea; 4 Graduate School of Medicine, Korea University, Seoul, Republic of Korea

## Abstract

The transmembrane nerve injury-induced protein 1 (Ninjurin1 or Ninj1) is involved in progressing inflammatory diseases. In this study, we aimed to investigate a novel function of Ninj1 in pulmonary fibrosis. We found that the expression of Ninj1 in a patient cohort was upregulated in the lung specimens of idiopathic pulmonary fibrosis patients as well as mice with bleomycin-induced pulmonary fibrosis. In addition, the BLM-injected Ninj1 KO mice exhibited a mild fibrotic phenotype, as compared to WT mice. Therefore, we hypothesized that Ninj1 would play an important role in the development of pulmonary fibrosis. We discovered that Ninj1 expression increased in BLM-treated macrophages and alveolar epithelial cells (AECs). Interestingly, macrophages bound to BLM-treated AECs were activated. However, when Ninj1 expression was suppressed in either of AECs or macrophages, contact-dependent activation of macrophages with AECs was diminished. In addition, introduction of recombinant mouse Ninj1^1–50^ to macrophages triggered an inflammatory response, but did not stimulate Ninj1-deficient macrophages. In conclusion, we propose that Ninj1 may contribute to activation of macrophages by enhancing interaction with AECs having elevated Ninj1 expression due to injury-inducing stimuli. Consequently, Ninj1 may be involved in the development of pulmonary fibrosis by enhancing inflammatory response of macrophages.

## Introduction

Idiopathic pulmonary fibrosis (IPF) is a chronic interstitial lung disease of unknown origin, characterized by irreversible and fatal progressive lung scarring‚ pulmonary dysfunction, and having no effective treatment^1,2^. The prognosis of IPF is extremely poor, with mean survival estimated to be 2 to 4 years after diagnosis^3–5^. In addition, the mortality of patients with lung cancer associated with IPF is significantly higher than patients with lung cancer alone^6^. However, even though previous studies have revealed several factors involved in the pathogenesis of IPF, the etiology is still poorly understood. Growing evidences suggest that pulmonary fibrosis is caused by a dysregulated wound healing process initiated by injury to the alveolar epithelial cells (AECs), and leading to a chronic inflammation^7,8^.

It has been reported that macrophage is a major inflammatory cell involved in the induction of pulmonary inflammation and fibrosis, by producing various pro-inflammatory and pro-fibrotic mediators^9–12^. The significance of macrophages is thoroughly discussed in a recent review, detailing the contribution of alveolar macrophages to lung diseases, and their importance as immune effector cells within the lung in patients with IPF^13^. In addition, the interaction between alveolar macrophages and AECs is an important factor in pulmonary inflammation and fibrosis, and contact-dependent effects of alveolar macrophages and AECs are required^14–17^.

Nerve injury induced protein 1 (Ninjurin1, or Ninj1) was first found in Schwann cells and its expression is induced, following an injury to the nerve^18^. Ninj1 consists of two transmembrane regions, an intracellular region, and extracellular region at the N- and C-termini. In addition, 12 amino acid residues (from Pro26 to Asn37) positioned in the extracellular region of N-terminus, were identified as a homophilic domain having binding affinity in a trans-interaction^19^. It was reported that Ninj1 mediates the transendothelial migration of immune cells such as monocytes, macrophages, and microglia in experimental autoimmune encephalopathy induced lesions^20^. Ifergan *et al*. also reported that Ninj1 in monocytes and dendritic cells enhances the extravasation in multiple sclerotic lesions of the human brain^21^. Recent reports indicate that Ninj1-expressing leukocytes adhere to the endothelial cells and/or other leukocytes via homophilic or heterophilic binding, and enter the site of inflammation or targeted tissues^22^. In addition, Ninj1 is also found to be up-regulated in B cells of acute lymphoblastic leukemia^23^ and multiple sclerosis^24,25^.

In this study, we hypothesized that Ninj1 could play a crucial role in developing pulmonary fibrosis, which is a chronic inflammatory disease. We demonstrated that Ninj1 deficiency ameliorated the bleomycin (BLM)-induced pulmonary fibrosis, which is the most commonly used pulmonary fibrosis model^26^. In addition, we show that Ninj1 enhances interaction between macrophages and AECs, leading to promotion of macrophage activation.

## Results

### Expression of Ninj1 is increased in developing pulmonary fibrosis

**Figure 1:**
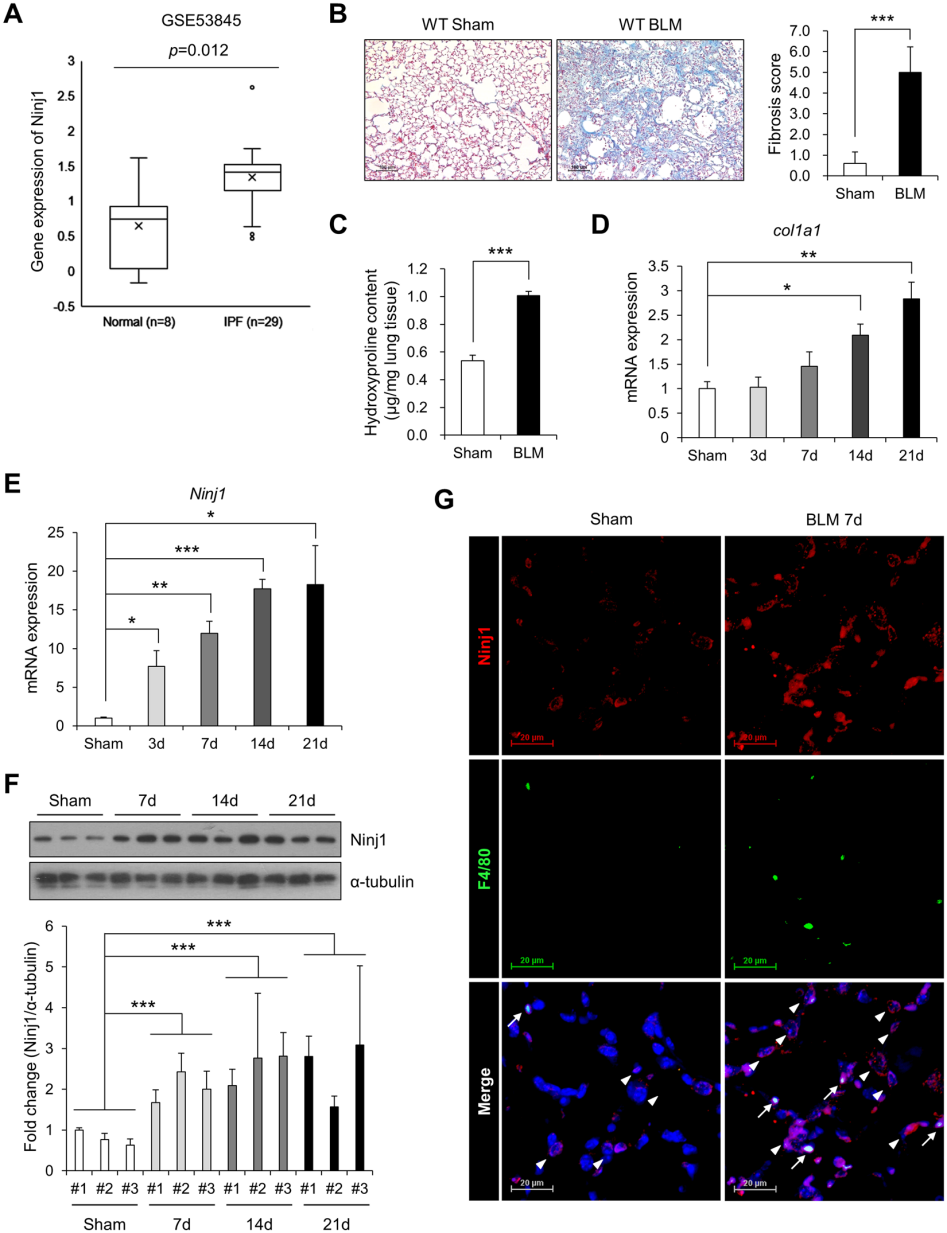
Expression of Ninj1 is increased in the fibrotic lungs. (**A**) Box plot for the expression of Ninj1 in the lung specimens of normal and IPF patients (n = 37) based on microarray gene expression data, GSE53845. (**B**) Representative images (left) of Masson’s trichrome staining (MTS) in lung sections of control and BLM-treated mice (at day 21, after saline or BLM injection, n = 5). Scale bar = 100 μm. Degree of fibrosis was scored according to the modified Ashcroft scoring system (n = 5, right). (**C**) The collagen contents accumulated in the lungs were quantified by hydroxyproline assay (n = 5). (**D**) Real-time PCR to determine *col1a1* mRNA expression levels, using lung specimen of control and BLM-treated mice at days 3, 7, 14 and 21 (n = 3). (**E**) Real-time PCR to determine *Ninj1* mRNA expression levels, using lung specimen of sham control and BLM-treated mice at days 3, 7, 14 and 21 (n = 3). (**F**) Western blot analysis to determine Ninj1 protein expression levels, using lung specimen of control and BLM-treated mice at days 7, 14 and 21 (n = 3). Representative images (upper) and semi-quantification of western blot (lower). (**G**) Representative images of immunohistochemistry for Ninj1 and F4/80 using paraffin block sections of lungs collected at day 7 after saline or BLM injection (n = 3). Arrows, F4/80^+^/Ninj1^+^ macrophages; arrow heads, F4/80^−^/Ninj1^+^ cells. Scale bar = 20 μm. The numerical data are expressed in means ± SD of triplicates. Semi-quantitative real-time PCR data are expressed in means ± SEM of triplicates. *p < 0.05; **p < 0.01; ***p < 0.001.

In order to determine if Ninj1 plays a role in developing pulmonary fibrosis, we first examined the expression of Ninj1 in the lung specimens from normal people (n = 8) and IPF patients (n = 29). In GSE53845, Ninj1 gene expression level was upregulated in fibrotic lungs (Fig. 1A). We also investigated Ninj1 expression level in BLM-induced pulmonary fibrosis model. Observation on Masson’s Trichrome Staining (MTS) revealed that collagens were accumulated in the lung tissue collected at day 21 after a single injection of BLM (Fig. 1B). Fibrosis index indicated that pulmonary fibrosis has been developed by BLM (Fig. 1B). In addition, hydroxyproline assay showed that the amount of collagens accumulated in the lungs at day 21 significantly increased (Fig. 1C) and *col1a1* mRNA expression, which encodes for type I collagen, was also elevated in a time-dependent manner (Fig. 1D). Interestingly, as pulmonary fibrosis was induced, the expression levels of Ninj1 mRNA (Figs 1E and S1A) and protein (Fig. 1F) were markedly elevated after BLM injection. Since Ninj1 is expressed in inflammatory cells such as macrophages^21,27^, we examined if elevation of Ninj1 expression in BLM-treated lungs was due to infiltration of Ninj1-expressing macrophages or increased Ninj1 expression in other cell types. Immunohistochemical analysis revealed that the number of Ninj1-expressing F4/80^+^ macrophages increased at day 7 after BLM treatment (Figs 1G and S1B, arrows). In addition, the expression of Ninj1 increased also in F4/80^−^ cells, such as AECs (Figs 1G and S1B, arrow heads). These results showed that when pulmonary fibrosis is induced by BLM, Ninj1 expressing-macrophages are infiltrated and the expression of Ninj1 is elevated in AECs, suggesting that Ninj1 may play a role in the development of pulmonary fibrosis.

### Ninj1 KO mice exhibit a mild fibrosis phenotype after administration of BLM

**Figure 2:**
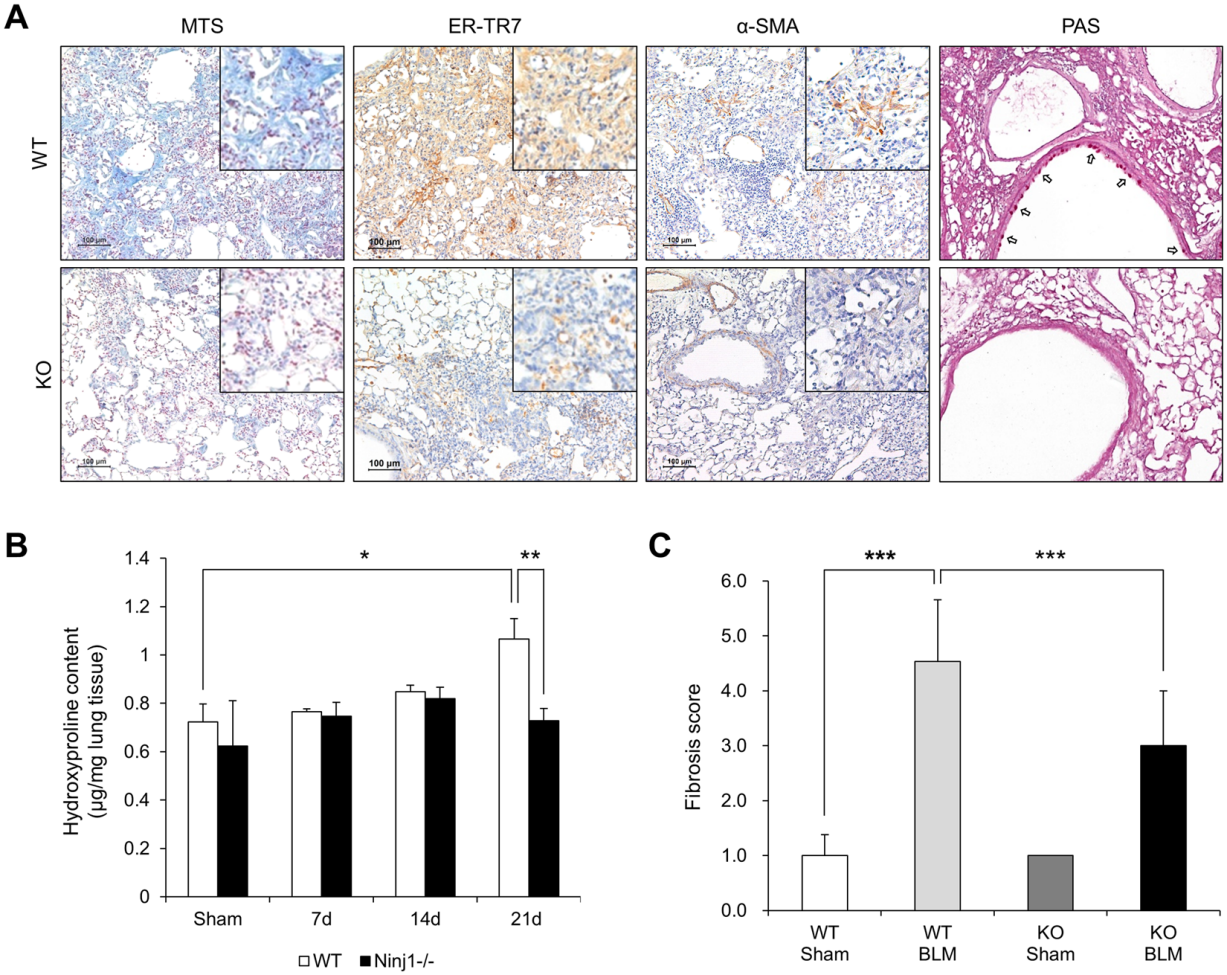
Pulmonary fibrosis was attenuated in BLM-treated Ninj1 KO mice. BLM was intratracheally injected to WT and Ninj1 KO mice, and necropsy was conducted at day 21 after injection. (**A**) Representative images of Masson’s trichrome staining (MTS, n = 13), immunohistochemistry for ER-TR7 (fibroblast marker) and α-SMA (myofibroblast marker) (n = 5), and periodic acid Schiff (PAS) staining (n = 5). Scale bar = 100 μm. (**B**) Hydroxyproline assay (n = 5). (**C**) Degree of fibrosis was scored according to modified Ashcroft scoring system (n = 13). All numerical data are expressed in means ± SD of triplicates. *p < 0.05; **p < 0.01; ***p < 0.001. ns = not significant.

As described in Fig. 1, BLM treatment led to elevation of Ninj1 expression by infiltrated macrophages and AECs. Therefore, hypothesizing that Ninj1 may contribute to the development of pulmonary fibrosis, we compared the development of pulmonary fibrosis between WT and Ninj1 KO mice. BLM (1 mg/kg) was intratracheally injected into WT and Ninj1 KO mice and lungs were collected at the indicated time points in Fig. 2 for further experiments. MTS and hydroxyproline assay showed collagen accumulation was significantly diminished in the lungs of Ninj1 KO mice at day 21, compared to those of WT mice (Fig. 2A,B). The mRNA expression of *col1a1* was also significantly lower in Ninj1 KO mice at day 14 and 21 (Supplementary Fig. S2A). In addition, the degree of fibrosis was also lower in Ninj1 KO mice than WT mice (Fig. 2C). One of the major aspects in the pathogenesis of pulmonary fibrosis is the activation of lung fibroblasts, whose proliferation increases and which are transformed into myofibroblasts^28^. Lung sections were subjected immunohistochemistry using antibodies against ER-TR7 as a fibroblast marker and α-SMA as a myofibroblast marker. We observed that accumulation of fibroblasts and myofibroblasts decreased in the lungs of Ninj1 KO mice, compared to WT mice (Fig. 2A). In addition, while mRNA expression *of α-SMA* in the lungs of WT mice gradually but significantly increased, Ninj1 KO mice exhibited only a slight increase in *α-SMA* expression (Supplementary Fig. S2B). These results show that activation of fibroblast by BLM instillation was diminished in Ninj1 KO mice. Moreover, PAS staining showed that secretion of mucin was less in the bronchus of Ninj1 KO mice than in WT mice (Fig. 2A) and mRNA expression of *MUC5B*, which encodes for tracheobronchial mucin, was also significantly lower in Ninj1 KO mice than in WT mice (Supplementary Fig. S2C), indicating that the degree of inflammatory status was lower in the lungs of Ninj1 KO mice than WT mice. Once again, these results suggested that Ninj1 would play an important role in developing pulmonary inflammation and fibrosis induced by BLM.

### BAL cell population does not alter between BLM-treated WT and Ninj1 KO mice

**Figure 3:**
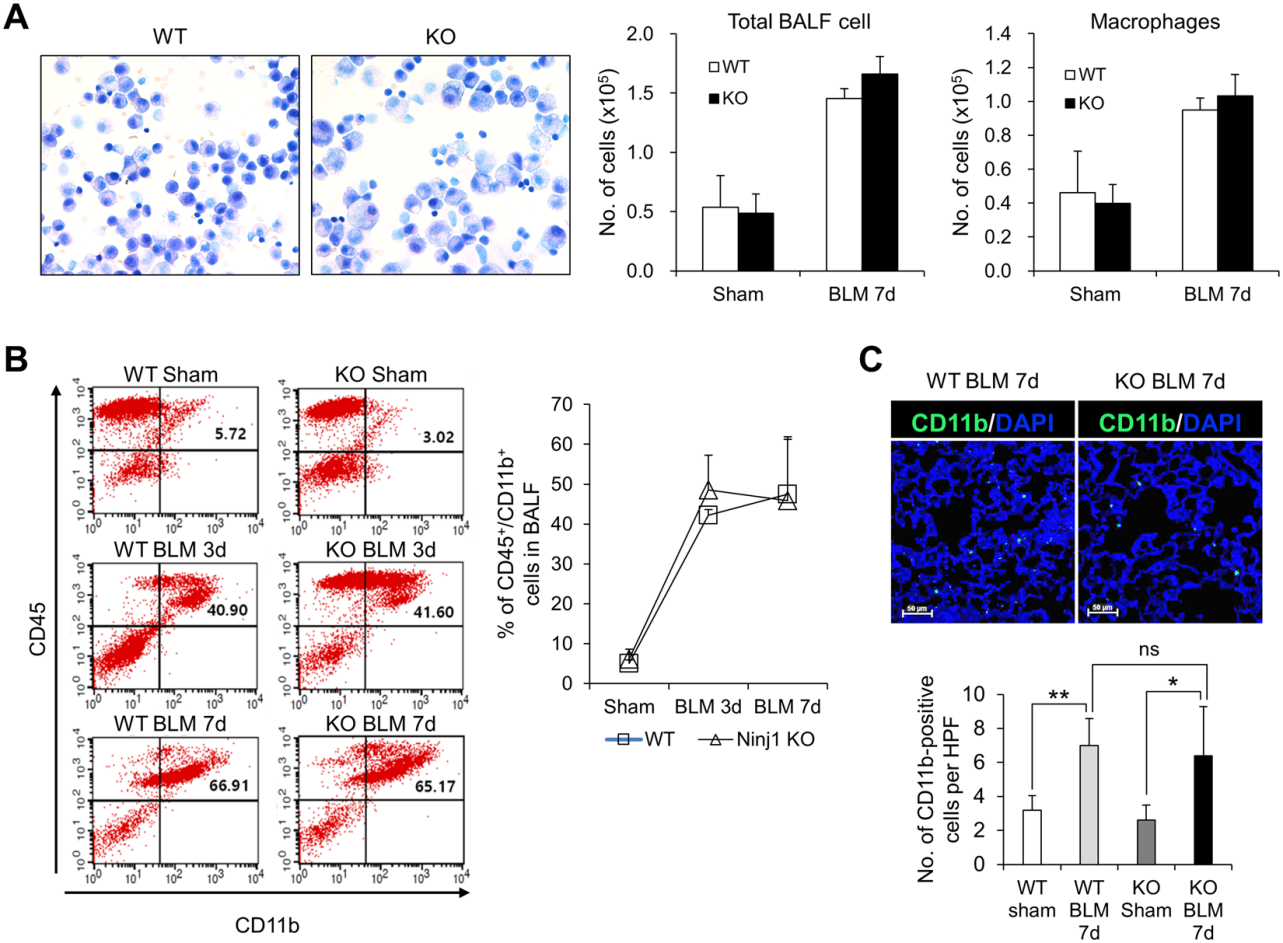
Inflammatory cell population does not alter between WT and Ninj1 KO mice after BLM instillation. (**A**) Representative images of BALF cells with Diff-Quick staining (left) (n = 5), total number of BALF cells from control and BLM-treated WT and Ninj1 KO mice (middle) (n = 5), and number of macrophages through Diff-Quick staining (n = 5). (**B**) Representative images (left) and quantification (right) of flow cytometry for CD45^+^/CD11b^+^ macrophages (n = 4). (**C**) Immunofluorescence assay for CD11b^+^ macrophages using frozen section of lungs (n = 5). Representative images (upper) and quantification (lower) for CD11b^+^ macrophages. HPF is abbreviation of high power field. Scale bar = 50 μm. All numerical data are expressed in means ± SD. *p < 0.05; **p < 0.01. ns = not significant.

During development of pulmonary fibrosis, inflammatory cells are recruited to injury site and produce various pro-inflammatory and pro-fibrotic mediators^28,29^. Therefore, we further examined if the fibrotic differences between WT and Ninj1 KO mice may have been resulted from differences in the recruitment of inflammatory cells. We observed that total number of BAL cells and macrophages increased in both WT and Ninj1 KO mice as BLM was injected (Fig. 3A). Unexpectedly, Diff-Quick staining in BALF showed that there was no significant difference in the number of total BAL cells between WT and Ninj1 KO mice, as well as in the number of macrophages (Fig. 3A). In addition, CD11b-positive macrophage population in BALF was not significantly different between the BLM-treated WT and Ninj1 KO mice (Fig. 3B,C). The population of inflammatory cells in the whole lungs was also assessed using cells from digested lungs. FACS analysis using anti-F4/80 (a macrophage marker), anti-CD3 (a T-cell marker) and anti-CD19 (B-cell marker) antibodies also showed no significant difference in population of those cell types infiltrated into lungs between WT and Ninj1 KO mice (Supplementary Fig. S3). These results indicated that even though there is a difference in fibrotic phenotype between BLM-treated WT and Ninj1 KO mice, the inflammatory cell population is not significantly different between them.

### Pro-inflammatory and fibrotic mediators are diminished in the lungs of Ninj1 KO mice

**Figure 4:**
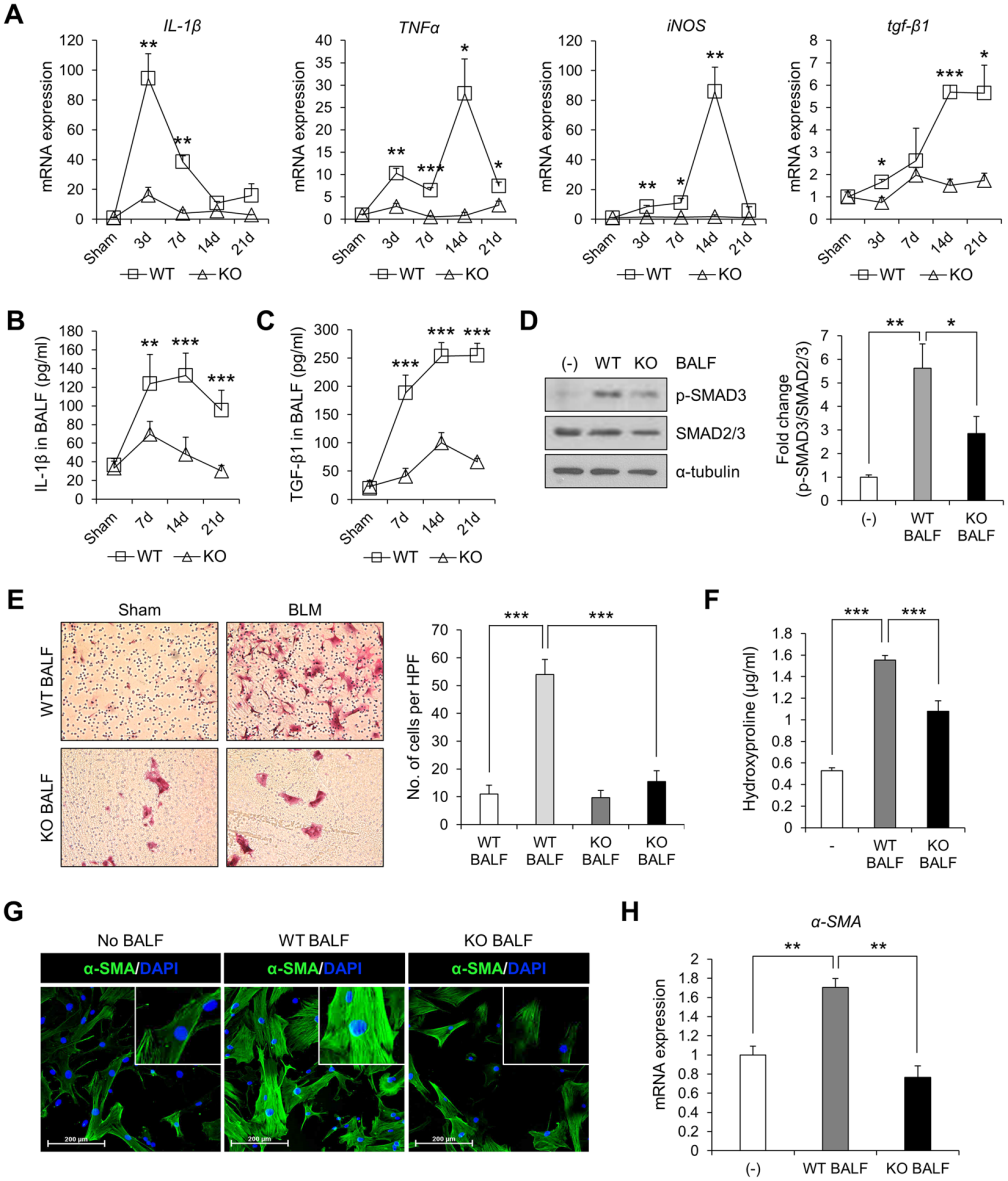
Expression of pro-inflammatory cytokines are diminished in BLM-treated Ninj1 KO mice. (**A**) Semi-quantitative real-time PCR to assess expression of pro-inflammatory and pro-fibrotic cytokines, *IL-1β*, *TNFα*, *iNOS* and *tgf-β1*, in the lungs of sham and BLM-treated WT and Ninj1 KO mice at day 3, 7, 14 and 21 (n = 3). (**B**) ELISA for IL-1β expression in BALF collected at days 7, 14, 21 after BLM injection (n = 3). (**C**) ELISA for TGF-β1 expression in BALF collected at days 7, 14, 21 after BLM injection (n = 3). (**D**–**H**) Effect of BALF from control and BLM-treated WT and Ninj1 KO mice at day 7 on activation of primary fibroblasts. (**D**) Western blot analysis to assess phosphorylation of SMAD3. Representative image (left) and semi-quantification (right) of western blot. (**E**) Transwell migration assay for BALF-treated primary fibroblasts. Representative images (left) and quantification (right) of migrated primary fibroblasts. (**F**) Hydroxyproline assay to measure collagen contents. (**G**) Immunofluorescence assay for α-SMA. (**H**) Semi-quantitative real-time PCR for α-SMA. The results of *in vitro* experiments (**D**–**H**) are representative of three independent experiments. The numerical data are expressed in means ± SD of triplicates. Semi-quantitative real-time PCR data are expressed in means ± SEM of triplicates. *p < 0.05, **p < 0.01; ***p < 0.001.

In the pathogenesis of pulmonary fibrosis, expression and secretion of pro-inflammatory and pro-fibrotic mediators such as IL-1β, TNFα and TGF-β1 play a crucial role^28,30^. As compared with control mice, while the mRNA expression of pro-inflammatory mediators (IL-1β, TNFα and iNOS) and pro-fibrotic cytokine (TGF-β1) was significantly elevated in the lungs of BLM-injected WT mice, the expression of these mediators in the lungs of Ninj1 KO mice was slightly induced but significantly less than WT mice (Fig. 4A). In detail, the average mRNA expression of *IL-1β* in WT lungs reached highest level at day 3, *TNFα* and *iNOS* at day 14, and *tgf-β1* at day 14 and 21 (Fig. 4A). ELISA also revealed that the level of secreted IL-1β and TGF-β1 in bronchoalveolar lavage fluid (BALF) of BLM-injected WT mice was significantly greater than BLM-injected Ninj1 KO mice (Fig. 4B,C). Since it has been reported that TGF-β1 signaling plays a central role in fibrogenesis via activation of fibroblasts^31^, we examined if BALF from WT or Ninj1 KO mice activates fibroblasts. Western blot analysis indicated that while BALF of BLM-treated WT mice induced phosphorylation of SMAD3, BALF of BLM-treated Ninj1 KO mice did not induce SMAD3 phosphorylation (Fig. 4D). The treatment of BALF from WT mice induced nuclear translocalization of SMAD3 in the primary fibroblasts, but BALF from the Ninj1 KO mice did not induce (Supplementary Fig. S4). In addition, migration of Ninj1 KO BALF-treated fibroblasts was significantly lower than the WT BALF-treated fibroblasts (Fig. 4E). Immunofluorescence (IF) assay for α-SMA expression revealed that WT BALF induced differentiation of fibroblast into myofibroblast, but no differentiation was observed with Ninj1 KO BALF (Fig. 4G). The mRNA expression of *α-SMA* was also significantly lower in Ninj1 KO BALF-treated fibroblasts (Fig. 4H). Finally, the hydroxyproline assay showed that collagen expression was diminished in Ninj1 KO BALF-treated fibroblasts (Fig. 4F). These results suggested that attenuation of pulmonary fibrosis in Ninj1 KO mice would have been due to changes in the pro-inflammatory and pro-fibrotic mediators, and Ninj1 could be one of the key molecules involved in production of the mediators.

### Altered expression of Ninj1 does not affect BLM-induced response in macrophages and AECs

As described in Fig. 1, injecting BLM resulted in the elevation of Ninj1 expression in the lungs of treated mice. Macrophage is a key inflammatory cell type in the pathogenesis of fibrosis^9,10,12^. Therefore, we examined for increase in expression of Ninj1 in macrophages due to inflammatory stimuli, namely BLM. We found that the expression of Ninj1 protein in Raw264.7 cells was up-regulated by BLM exposure in a dose-dependent manner (Supplementary Fig. S5A). FACS analysis also indicated that the expression of surface Ninj1 was increased by BLM treatment (Supplementary Fig. S5B). Next, the expression of Ninj1 was examined in AECs, which has been reported to play an important role in pulmonary fibrosis^2,32^. Interestingly, the expression level of total and surface Ninj1 in MLE-12 cells, a type II pneumocyte cell line, also increased (Supplementary Fig. S5C,D). These results suggested that Ninj1 may play a pro-fibrotic role in macrophages and AECs during fibrogenesis.

**Figure 5:**
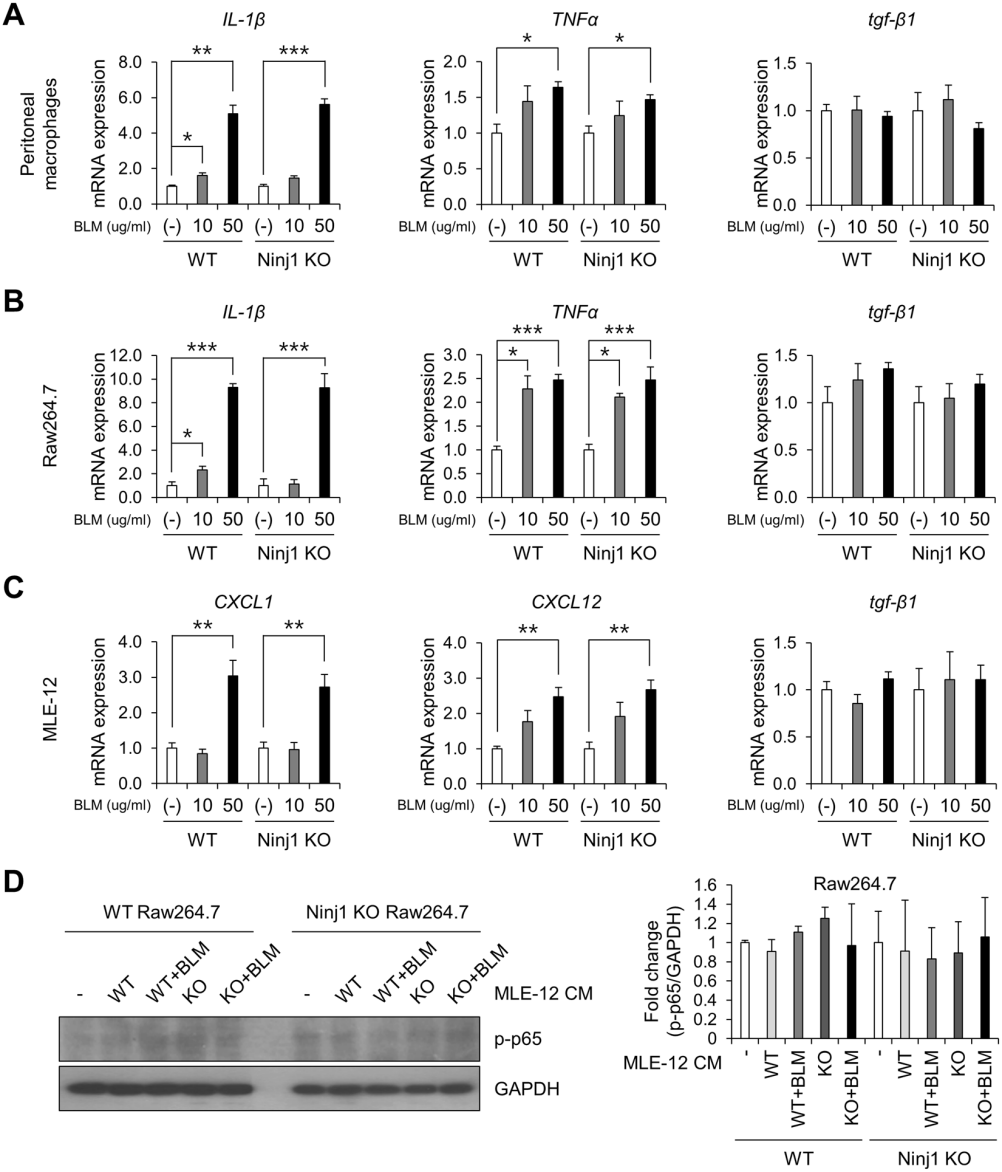
Ninj1 deficiency does not alter the production of inflammatory cytokines. Semi-quantitative real-time PCR to assess the expression of cytokines, *IL-1β*, *TNFα*, *iNOS* and *tgf-β1*, in WT and Ninj1 KO peritoneal macrophages (**A**) or WT and Ninj1 KO Raw264.7 cells (**B**) treated with 0, 10 or 50 μg/ml of BLM for 24 hours. (**C**) Semi-quantitative real-time PCR to determine expression of inflammatory mediators in WT and Ninj1 KO MLE-12. (**D**) Western blot analysis to examine activation of p65 in Raw264.7 cells treated with CM from MLE-12 in various conditions, as indicated in the figure. Representative image (left) and semi-quantification (right) of western blot. The results are representative of three independent experiments. Semi-quantitative real-time PCR data are expressed in means ± SEM of triplicates. *p < 0.05; **p < 0.01; ***p < 0.001.

Since the expression of Ninj1 was elevated in both macrophages and ACEs, we then examined if Ninj1 deficiency alters inflammatory and fibrotic response in macrophages and AECs. We investigated the differences in BLM-induced response between peritoneal macrophages from WT and Ninj1 KO mice. Unexpectedly, there was no significant difference in the expression of cytokines (*IL-1β*, *TNFα* and *tgf-β1*) between WT and Ninj1 KO peritoneal macrophages (Figs 5A and S6A). We further assessed the response to BLM in WT and Ninj1 KO Raw264.7 cell lines and found that there was no significant difference in the expression of cytokines depending on Ninj1 expression (Figs 5B and S6B). Next, the previous studies reported that AECs play a crucial role in recruitment of macrophages in lung inflammatory diseases by producing pro-inflammatory mediators^33,34^. We therefore examined if the expression of pro-inflammatory mediators was reduced in Ninj1-deficient AECs. The mRNA analysis showed the expression of pro-inflammatory mediators, *CXCL1*, *CXCL12* and *tgf- β1*, was not markedly different between WT and Ninj1 KO MLE-12 (Fig. 5C). Consistent with these results, when the conditioned media (CM) from WT or Ninj1 KO MLE-12 cells, with or without BLM, was introduced to WT and Ninj1 KO Raw264.7 cells, activation of p65 remained unaltered (Fig. 5D). These results indicated that Ninj1 deficiency did not affect the response of macrophages and AECs to extracellular stimuli.

### Ninj1 Deficiency does not affect cell-to-cell adhesion between macrophages and AECs

As previously mentioned, the interaction between macrophages and alveolar epithelial cells is an essential process in pulmonary inflammatory diseases^14–16^. In addition, it has been reported that Ninj1 has homophilic binding properties for its cell-to-cell adhesion^22^. Therefore, we examined if Ninj1 was involved in cell-to-cell adhesion between AECs and macrophages. By FACS analysis, we observed that Raw264.7 cells bound to WT MLE-12 cells were increased after exposure to BLM (Supplementary Fig. S7A). However, unexpectedly, the number of Raw264.7 cells bound to Ninj1 KO MLE-12 cells was also increased due to BLM exposure (Supplementary Fig. S7B). These results suggested that Ninj1 may not be necessary for adhesion of two cell lines.

### Ninj1 plays a crucial role in stimulating macrophages by enhancing interaction with AECs

**Figure 6:**
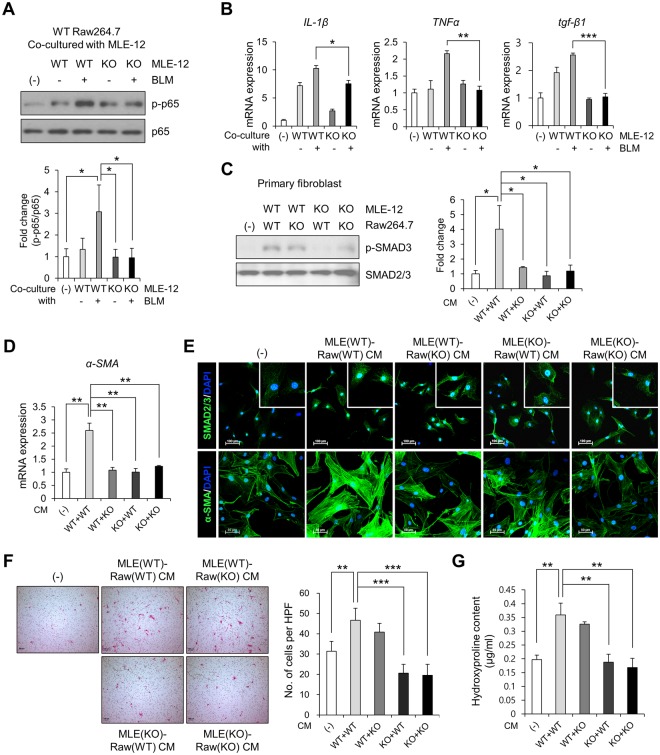
Ninj1 is involved in the interaction between MLE-12 and Raw264.7 cells. (**A**,**B**) WT Raw264.7 cells were co-cultured with WT or Ninj1 KO MLE-12 cells and inflammatory response of Raw264.7 cells were assessed. (**A**) Western blot analysis to assess phosphorylation of p65 in WT or Ninj1 KO Raw264.7 cells after co-culture with BLM (50 μg/ml)-treated or untreated WT or Ninj1 KO MLE-12. Representative image (upper) and semi-quantification (lower) of western blot. (**B**) Semi-quantitative real-time PCR for cytokines in WT or Ninj1 KO Raw264.7 cells after co-culture with BLM (50 μg/ml)-treated or untreated WT or Ninj1 KO MLE-12. (**C**–**G**) Assessment of primary fibroblast activation by CM from co-culture of WT or Ninj1 KO Raw264.7 cells, and WT or Ninj1 KO MLE-12 cells. (**C**) Western blot analysis for SMAD3 phosphorylation after CM treatment. Representative image (left) and semi-quantification (right) of western blot. (**D**) Semi-quantitative real-time PCR for α-SMA mRNA expression. (**E**) Immunofluorescence assay for α-SMA expression and SMAD3 nuclear localization. (**F**) Transwell migration assay. Representative images (left) and quantification (right) of migrated primary fibroblasts. (**G**) Hydroxyproline assay to measure collagen contents secreted by primary fibroblasts. The results are representative of three independent experiments. The numerical values are expressed in means ± SD of triplicates. Semi-quantitative real-time PCR data are expressed in means ± SEM of triplicates. *p < 0.05; **p < 0.01; ***p < 0.001. (Abbreviations: WT MLE-12 + WT Raw264.7, WT + WT; WT MLE-12 + Ninj1 KO Raw264.7, WT + KO; Ninj1 KO MLE-12 + WT Raw264.7, KO + WT; Ninj1 KO MLE-12 + Ninj1 KO Raw264.7, KO + KO).

We then hypothesized that Ninj1 on lung epithelial cells contributes to activation of macrophages, which is triggered by binding of macrophages to AECs. We observed that Raw264.7 cells bound to WT MLE-12 cells exhibited activation of the inflammatory signaling protein, p65 (Fig. 6A). Moreover, in Raw264.7 cells bound to the BLM-treated WT MLE-12 cells, there was an increased activation of p65 (Fig. 6A). Surprisingly, even though p65 was activated when bound to Ninj1 KO MLE-12 cells, no further increase was observed in the activation when bound to BLM-treated Ninj1 KO MLE-12 cells (Fig. 6A). Consistent with these results, while the expression of IL-1β, TNFα and TGF-β1 increased when Raw264.7 cells were bound to BLM-treated WT MLE-12, the expression of cytokines was observed to be diminished in the Raw 264.7 cells bound to BLM-treated Ninj1 KO MLE-12 (Fig. 6B). We further examined if the CM from co-culture of MLE-12 and Raw264.7 cells could activate fibroblasts, and if Ninj1 deficiency in these two cell lines altered the activation of fibroblasts. As shown in Fig. 6C,D, when MLE(WT)-Raw(WT)CM or MLE(WT)-Raw(KO) CM was introduced to primary fibroblasts, phosphorylation of SMAD3 was induced (Fig. 6C), and SMAD3 was translocated into the nucleus. However, MLE(KO)-Raw(WT) CM or MLE(KO)-Raw(KO) CM did not induce phosphorylation and nuclear localization of SMAD3 (Fig. 6C,E). The mRNA and protein expression of α-SMA in fibroblasts was significantly lower when Ninj1 was deficient in either of MLE-12 or Raw264.7 cell line, compared to fibroblasts treated with MLE(WT)-Raw(WT) CM (Fig. 6D,E). In addition, it was observed that migration of fibroblasts was markedly decreased when Ninj1 was deficient in either cell line (Fig. 6F). The hydroxyproline assay showed that production of collagens in fibroblasts was also reduced when Ninj1 was deficient in either of the cell lines (Fig. 6G). These results suggested that when macrophages are bound to AECs, they are activated and produce pro-fibrotic mediators, leading to activation of fibroblasts. However, the results also suggested that if Ninj1 is deficient either in macrophages or AECs, macrophages are not activated even though they are bound to AECs.

### rmNinj1^1–50^ enhances inflammatory response in macrophages

**Figure 7:**
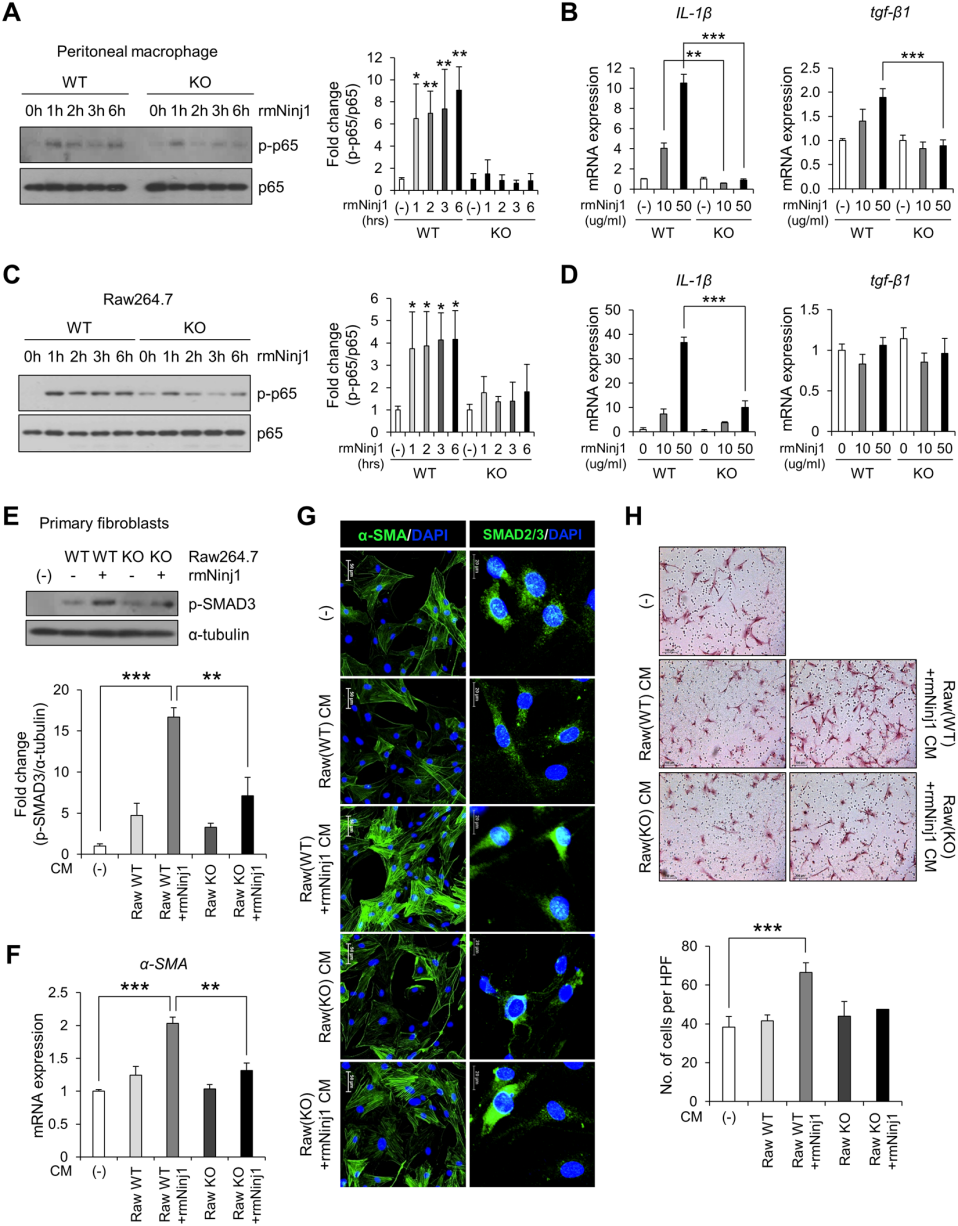
rmNinj1^1–50^ (rmNinj1) activates WT macrophages but not Ninj1 KO macrophages. Western blot analysis to assess the phosphorylation of p65 in rmNinj1^1–50^ (50 μg/ml)-treated WT or Ninj1 KO peritoneal macrophages (**A**), and WT or Ninj1 KO Raw264.7 cells (**C**). (**A**,**C**) Representative image (left) and semi-quantification (right) of western blot. Semi-quantitative real-time PCR for expression of *IL-1β* and *tgf-β1* in rmNinj1^1–50^ (50 μg/ml)-treated WT or Ninj1 KO peritoneal macrophages (**B**), and WT or Ninj1 KO Raw264.7 cells (**D**). (**E**–**H**) Assessment of primary fibroblast activation by CM from WT or Ninj1 KO Raw264.7 cells with or without rmNinj1^1–50^ (50 μg/ml) treatment. (**E**) Western blot analysis for SMAD3 phosphorylation. Representative image (upper) and semi-quantification (lower) of western blot. (F) Semi-quantitative real-time PCR for expression of *α-SMA*. (**G**) Immunofluorescence assay for α-SMA expression and SMAD3 nuclear localization. (**H**) Transwell migration assay. Representative images of migrated primary fibroblasts (upper) and quantification of migrated cells (lower). The results are representative of three independent experiments. The numerical values are means ± SD. Semi-quantitative real-time PCR data are expressed in means ± SEM of triplicates. *p < 0.05; **p < 0.01; ***p < 0.001. ns = not significant.

As interaction between MLE-12 and Raw264.7 cells induces the production of pro-fibrotic cytokines and Ninj1 deficiency attenuates the effect, we hypothesized that Ninj1 directly activates macrophages. We generated recombinant mouse Ninj1^1-50^ a.a. (rmNinj1^1–50^ or rmNinj1), which covers the extracellular domain of Ninj1 where the homophilic binding domain is located^19^. Peritoneal macrophages from WT and Ninj1 KO mice were first treated with rmNinj1^1–50^ and the inflammatory response was evaluated. Interestingly, we observed that the phosphorylation of NF-kB signaling protein p65 was significantly elevated in WT Raw264.7 cells and peritoneal macrophages when exposed to rmNinj1^1–50^ (Fig. 7A,C). On the other hand, the exposure of rmNinj1^1–50^ to Ninj1 KO macrophages slightly induced phosphorylation of p65 at 1 hour but the effect was not preserved after 1 hour (Fig. 7A,C). In addition, the mRNA expression of *IL-1β* was induced in WT macrophages by rmNinj1^1–50^ exposure while its expression was not increased in Ninj1 deficient conditions (Fig. 7B,D). Incidentally, while the expression of *tgf-β1* in WT peritoneal macrophages was increased by rmNinj1^1–50^ exposure, the expression of *TGF-β1* remained unchanged in WT Raw264.7 cells (Fig. 7B,D). The expression of *TGF-β1* also remained unchanged in both Ninj1 KO peritoneal macrophages and Raw264.7 cells (Fig. 7B,D). In order to confirm the effect of rmNinj1^1–50^ on macrophages, the CM was collected from rmNinj1^1–50^-treated WT or Ninj1 KO Raw264.7 cells and administered to primary fibroblasts. As expected, the CM from rmNinj1^1–50^-treated WT Raw264.7 cells induced the phosphorylation of SMAD3 in fibroblasts, which was not observed in fibroblasts treated with CM from rmNinj1^1–50^-treated Ninj1 KO Raw264.7 cells (Fig. 7E). Also, the nuclear localization of SMAD3 was decreased in fibroblasts treated with CM from rmNinj1^1–50^-treated Ninj1 KO Raw264.7 cells (Fig. 7F). Furthermore, the expression of *α-SMA* was decreased in fibroblasts treated with CM from rmNinj1^1–50^-treated Ninj1 KO Raw264.7 cells, compared to CM from WT Raw264.7 cells (Fig. 7G). Migration of fibroblasts treated with CM from rmNinj1^1–50^-treated Ninj1 KO Raw264.7 cells was also reduced (Fig. 7H). The rmNinj1^1–50^ was also introduced to MLE-12 cells in order to assess activation of AECs, which would produce pro-inflammatory and pro-fibrotic cytokines. However, mRNA expression of *CXCL1*, *CXCL12* and *tgf-β1* was not induced by rmNinj1^1–50^ (Supplementary Fig. S8). These results suggested that Ninj1 would directly activate macrophages, but not AECs, possibly through homophilic binding of Ninj1, resulting in the production of pro-fibrotic proteins that activate fibroblasts.

## Discussion

Recent studies have emphasized the role of Ninj1 in induction of inflammation^21,22,27,35^. Here, we further demonstrated a novel role of Ninj1 during the development of pulmonary fibrosis. This study revealed the involvement of Ninj1 in stimulation of inflammatory response in macrophages by promoting contact-dependent interaction with AECs, thereby proving a contribution in developing pulmonary fibrosis.

In this study, we first found that following BLM injection, the histology of the lungs from Ninj1 KO mice exhibited mild inflammation and fibrotic phenotype, as compared to the WT mice. One aspect in the pathogenesis of pulmonary fibrosis is an increase in the infiltration of immune cells including macrophages, thereby leading to chronic inflammation^12,28,29^. Ninj1 has been reported to have a role in the migration of immune cells^21^. Unexpectedly, the number of infiltrated inflammatory cells in the lungs of BLM-treated Ninj1 KO mice was not significantly different from BLM-treated WT mice. Conversely, the expression of pro-inflammatory and pro-fibrotic mediators were diminished in the lungs of Ninj1 KO mice. These results suggested that Ninj1 plays a role in the induction of inflammatory response during the development of pulmonary fibrosis.

We then investigated how Ninj1 deficiency reduced production of inflammatory cytokines. Recent reports indicated that macrophages are the major inflammatory cell type in the development of pulmonary fibrosis^8^. When the lungs are injured, macrophages are activated and release pro-inflammatory cytokines (IL-1β and TNFα) as well as a pro-fibrotic mediator (TGF-β1) which directly activate the fibroblasts to release numerous ECM components^11,36,37^. It was reported that Ninj1 is expressed mainly in macrophages and plays a crucial role in regulating the activity of macrophages^38^. Since Ninj1 expression was elevated in macrophages by BLM in our observation, we hypothesized that Ninj1 deficiency may reduce inflammatory response in macrophages. Unexpectedly, Ninj1 deficiency did not alter the inflammatory response of macrophages to BLM. Histological analysis and *in vitro* experiment revealed that Ninj1 expression was upregulated also in AECs by BLM treatment. Since alveolar epithelial cells paly a pivotal role in the development of pulmonary fibrosis^2,32,39^, we examined if Ninj1 deficiency alters BLM-induced damage response in AECs. However, we observed that even though the expression of Ninj1 in AECs was elevated by BLM treatment, there was no significant difference in the expression of inflammation-stimulating factors between control and Ninj1-deficient AECs. These results suggested that even though BLM enhances Ninj1 expression, inflammatory response by BLM does not require Ninj1. The further study is necessary to investigate how BLM promoted Ninj1 expression.

We then hypothesized that Ninj1 would be involved in cell-to-cell contact interaction by homophilic binding. The previous reports indicated that contact-dependent interaction between AECs and macrophages is essential for initiation of inflammation in the pathogenesis of interstitial lung diseases^14–16^. Ninj1 is a homomeric adhesion molecule, which leads to cell-to-cell adhesion^22,24^. However, our data demonstrated that there was no significant difference in adhesion of AECs and macrophages depending on Ninj1 expression. Interestingly, we found that, macrophages were activated as they were bound to AECs and they were activated more when bound to BLM-treated AECs, leading to a significant increase in production of pro-fibrotic mediators that activates fibroblasts. In addition, even though macrophages were bound to AECs, they are not activated when Ninj1 is depleted either in AECs and macrophages. In this study, we confirmed that Ninj1-mediated interaction between AECS and macrophages enhanced activation of macrophages so that they produced pro-fibrotic mediators, which finally led to myofibroblast differentiation. Even though Ninj1 was not required for adhesion of AECs and macrophages as we concluded, macrophages would be activated as Ninj1 promoted a contact-dependent interaction with AECs. Therefore, we hypothesized that Ninj1 may directly activate macrophages. Surprisingly, inflammatory response was induced when WT macrophages were treated with rmNinj1^1–50^, whereas the Ninj1 KO macrophages were not activated by rmNinj1^1–50^. However, while IL-1β expression level increased, TGF-β1 expression level did not increase. In addition, even though the expression of TGF-β1 was not up-regulated in macrophages, their CM induced SMAD3 activation. IL-1β signaling, which is one of the major inflammatory signaling pathways, is involved in gene expression of TGF-β1 which would transmit autocrine signal to activate fibroblast^40^. Taking these data together, since rmNinj1^1–50^ includes N-terminal region where the homophilic binding domain is lcoated^19,20^, it may have directly activated macrophages by interacting with Ninj1 on macrophages. However, the molecular mechanism of how Ninj1 on AECs enhances the inflammatory response of macrophages needs further investigation.

To summarize our results, we have investigated a novel function of Ninj1 in pulmonary fibrosis. We found that the expression of Ninj1 in lungswas elevated by BLM, and AECs and macrophages expressed increased Ninj1 level. The elevated Ninj1 expression enhanced the activation of macrophages by promoting the interaction with AECs, leading to increase in pro-fibrotic mediators that activate fibroblast. A recent report has suggested that targeting macrophage-directed factors could be an effective therapeutic strategy for patients with IPF^41^. Taking all together, our findings indicate that Ninj1 could be a potential target to inhibit activation of macrophages for preventing future episodes of IPF incidence.

## Materials and Methods

### Animal care and experiments

All animal experiments were approved by the Institutional Animal Care and Use Committee at the National Cancer Center and carried out in accordance with the relevant guidelines and regulations. Ninj1 KO mice were provided by GT Oh^20,42^. C57BL/6J WT and Ninj1 KO mice were maintained under 12 h light/dark cycles at 22 °C and 60% humidity, and provided with food and water *ad libitum*. Eight-wk-old WT and Ninj1 KO mice were used in this study. To induce pulmonary fibrosis, 1 mg/kg of BLM (Carbosynth, Compton, UK) or PBS was intratracheally injected, and necropsy was performed at 1, 3, 5, 7, 14 and 21 days after injection. Before excision of lungs, cell-free bronchoalveolar lavage fluid (BALF) and BAL cells were collected at day 3, 7 and 21. as described in the previous report^43^. The lungs collected at day 21 were fixed in 10% neutral formalin, and a paraffin block was generated. On days 1, 3, 5, 7, and 14, the right lungs were frozen in liquid nitrogen for RNA and protein analysis, whereas the left lungs were fixed in 10% neutral formalin for histological analysis. To measure levels of IL-1β and TGF-β1, enzyme-linked immunosorbent assay (ELISA kits; IL-1β, KOMA Biotech, Seoul, Korea; TGF-β1, AbFrontier, Seoul, Korea) was conducted using frozen lung tissue and cell-free BALF collected at day 7, following manufacturer’s instruction.

### Extraction of microarray gene expression dataset

The dataset, GSE53845, was extracted to identify 8 normal and 29 IPF lung specimens. The data were analyzed as previously described^44^.

### Inflammatory cell analysis

In order to assess the inflammatory cell population in brochalveolar lavage (BAL) cells, 100 µL of the BAL cell suspension was fixed on slides using the Shandon Cytospin 4 Cytocentrifuge (Thermo Scientific, MA, USA). The cells were stained with Diff-Quick staining kit and a differential cell count was performed under light microscope. The remaining BAL cells were double-stained with fluorescent anti-CD45 and anti-CD11b antibodies. FACS analysis was performed using BD FACSCalibur (BD Bioscience). The antibodies and methods are described in Supplementary Materials and Methods.

Using lung tissue, the lungs were minced until the fragment size is smaller than 2 mm. The lung fragments were digested by incubating them with Type IV Collagenase at 37 °C for 1 hour. The digested cells were centrifuged and the pallet was washed with PBS 3 times. The lung cells were stained with fluorescent anti-CD45, anti-CD3, anti-CD19 and anti-F4/80 antibodies and FACS analysis was performed using BD FACSCalibur (BD Bioscience). The antibodies and methods are described in Supplementary Materials and Methods.

### Primary cell isolation

Lung primary cells were isolated from lungs of C57BL/6J mice and peritoneal macrophages were isolated from WT or Ninj1 KO mice as described in the previous report^45^. The detailed method is described in Supplementary Materials and Methods.

### Recombinant mouse Ninj1^1–50^

Recombinant protein was generated as described in our previous report^46^. The primers used to amplify the gene that encodes Ninj1 (1–50 a.a.) are described in Supplementary Materials and Methods

### Cell culture and treatment

All cell lines and primary cells were cultured at 37 °C in the humidified chamber with 5% CO_2_. Primary lung fibroblasts were isolated as described in Supplementary Materials and Methods. Raw264.7 was purchased from Korean Cell Line Bank (KCLB, Seoul, Korea). Raw264.7 and peritoneal macrophages were cultured in 10% FBS-supplemented DMEM (WelGene, Daegu, Korea) with streptomycin (100 µg/ml)/penicillin (100 units/ml). Raw264.7 cells and peritoneal macrophages were exposed to BLM (50 μg/ml) or recombinant mouse Ninj1 (rmNinj1, 0, 10 or 50 μg/ml) for 6 hours or various time periods as indicated in figures. The cells were harvested for western blot and RT-PCR. MLE-12, a pneumocyte cell line, was purchased from ATCC (Manassas, VA, USA). MLE-12 was cultured in 2% FBS-supplemented DMEM (WelGene, Daegu, Korea) with streptomycin (100 µg/ml)/penicillin (100 units/ml). MLE-12 cells were transfected with scramble or siNinj1 using TransIT-X2 System (Mirus Bio LLC., WI, USA), following manufacturer’s protocol and incubated for 48 hours. MLE-12 cells were treated with BLM (0, 10 or 50 µg/ml) or rmNinj1 (0, 10 or 50 µg/ml) for 24 hours, and western blot and RT-PCR were performed.

### Generation of Ninj1 KO cell lines using CRISPR Cas9

CRISPR Cas9 All-in-one lentiviral expression vectors targeting Ninj1 were purchased from transOMIC Technologies inc. (Huntsville, AL, USA). MLE-12 and Raw264.7 cells were transfected with the CRISPR Cas9 expression vector using TransIT®−2020 Transfection Reagent (Mirus Bio LLC), following manufacturer’s instruction. The transfected cells were sorted by tRFP, using SONY cell sorter SH800Z (SONY, Tokyo, Japan). The sorted cells were plated on 96-well plate for single cell colony selection. Ninj1 expression was evaluated by performing western blot analysis. Ninj1-expressing cells were referred to as WT and Cas9-drived Ninj1-deficient cells as Ninj1 KO.

### Co-culture of MLE-12 and Raw264.7

WT or Ninj1 KO MLE-12 cells with or without BLM (50 µg/ml) treatment for 12 hours and CFSE-stained WT or Ninj1 KO Raw264.7 cells were co-cultured for 30 min, and the number of Raw264.7 cells bound to MLE-12 cells was assessed using the BD FACSCalibur (BD Bioscience). For RNA and protein analysis, after co-culture of WT or Ninj1 KO MLE-12 and CFSE-stained WT or Ninj1 KO Raw264.7 for 6 hours, Raw264.7 cells were sorted using the SONY cell sorter SH800Z (SONY, Tokyo, Japan), and RNA and protein were isolated from the sorted Raw264.7 cells. To collect conditioned media (CM), the media was changed to serum-free medium after 6 hours of co-culture and CM was collected 12 hours after media change.

### Stimulation of Fibroblasts by BALF and CM

Primary fibroblasts were isolated from C57BL/6J. The detailed method is described in Supplementary Materials and Methods. In order to evaluate activation of fibroblasts, activation and localization of SMAD2/3, migration, production of collagens and expression of α-SMA were assessed. To examine activation and localization of SMAD2/3, BALF collected at day 21 (1:4 diluted in serum-free DMEM) or CM from co-culture of MLE-12 and Raw264.7 cells or rmNinj1-treated Raw264.7 cells were introduced after 24 hours of starvation. The fibroblasts were incubated for 30 min in a humidified CO_2_ incubator and harvested for western blotting and immunofluorescence assay. To assess migration of fibroblasts, the transwell migration assay was performed as described in our previous report^47^. Briefly, cell-free BALF collected from the BLM-treated WT and Ninj1 KO mice (day 7), or the CM from co-cultures of MLE-12 and Raw264.7 cells or rmNinj1-treated Raw264.7 cells was used as chemoattractant. The primary fibroblasts were seeded in the upper chamber of the transwell inserts and diluted BALF (1:4 dilutions in serum-free medium) or CM was placed in the lower chamber. The fibroblasts were incubated in a humidified CO_2_ incubator for 6 hours, and the number of migrated fibroblasts was counted under light microscope. To measure collagens secreted by primary fibroblasts, the fibroblasts were incubated with diluted BALF or CM for 12 hours. The media was collected and subjected to hydroxyproline assay. To examine expression of α-SMA, the fibroblasts were incubated with diluted BALF or CM for 12 hours and collected for RNA isolation and immunofluorescence assay. RNA was isolated and cDNA was synthesized, followed by real-time PCR.

### Histological evaluation of pulmonary fibrosis

To observe histological changes after BLM treatment, 5 µm-thick lungs tissue sections were stained with hematoxylin and eosin. Masson’s Trichrome Staining (MTS) was also performed to evaluate the severity of pulmonary fibrosis, according to modified Ashcroft scale described in the previous report^48^. Periodic acid schiff (PAS) staning was performed to evaluate inflammatory status in the fibrotic lungs, using 5 µm-thick lung tissue sections. Histology of lung tissue was observed under light microscope.

### Immunohistochemistry and immunofluorescence assay

Immunohistochemistry (IHC) and immunofluorescence assay (IF) were performed according to the protocol in our previous report^47^. The detailed method and the antibodies used are described in Supplementary Materials and Methods.

### Hydroxyproline assay

The amount of collagens accumulated in the lungs or secreted from primary fibroblasts were measured using the Hydroxyproline Colorimetric Assay Kit (BioVision, CA, USA), following the manufacturer’s instruction. The detailed method is described in Supplementary Materials and Methods.

### mRNA expression analysis

RNA was isolated from cells and lung specimens using TriZol (Invitrogen, CA, USA) and 2 µg of RNA was used to synthesize cDNA using PrimeScript RT reagent Kit (Takara, Shiga, Japan) as described in the manufacturer’s instruction. Reverse-transcription PCR (RT-PCR) and semiquantitative real-time PCR (qPCR) were performed, using the primers listed in Supplementary Materials and Methods. The RT-PCR products were detected through agarose gel electrophoresis. qPCR was proceeded, following the method in the previous report^49^. The detailed method for qPCR is described in Supplementary Materials and Methods.

### Western blot analysis

Western blot was performed as described in our previous report^47^. The antibodies and methods are described in Supplementary Materials and Methods.

### Statistical analysis

SPSS23.0 was used for all statistical analyses. Welch’s t-test was used to compare gene expression level between lungs from healthy and IPF patients. All experimental data were evaluated by Student’s t test. All graphical data were expressed as mean ± SD values of triplicate experiments. The significance level was limited to 5% (*p* < 0.05).

## Electronic supplementary material

Supplementary Information

## Data Availability

The authors declare that all other relevant data are available from the authors upon request.

## References

[1] Zeki AA (2010). Geoepidemiology of COPD and idiopathic pulmonary fibrosis. J Autoimmun.

[2] Selman M, Pardo A (2006). Role of epithelial cells in idiopathic pulmonary fibrosis: from innocent targets to serial killers. Proc Am Thorac Soc.

[3] Raghu G (2011). An official ATS/ERS/JRS/ALAT statement: idiopathic pulmonary fibrosis: evidence-based guidelines for diagnosis and management. Am J Respir Crit Care Med.

[4] American Thoracic Society (2000). Idiopathic pulmonary fibrosis: diagnosis and treatment. International consensus statement. American Thoracic Society (ATS), and the European Respiratory Society (ERS). Am J Respir Crit Care Med.

[5] Strock Stephen B, Alder Jonathan K, Kass Daniel J (2018). From bad to worse: when lung cancer complicates idiopathic pulmonary fibrosis. The Journal of Pathology.

[6] Lee T (2014). Lung cancer in patients with idiopathic pulmonary fibrosis: clinical characteristics and impact on survival. Respir Med.

[7] Selman M (2001). Idiopathic pulmonary fibrosis: prevailing and evolving hypotheses about its pathogenesis and implications for therapy. Ann Intern Med.

[8] King TE, Pardo A, Selman M (2011). Idiopathic pulmonary fibrosis. Lancet.

[9] Borthwick LA (2016). Macrophages are critical to the maintenance of IL-13-dependent lung inflammation and fibrosis. Mucosal Immunol.

[10] Wynn TA (2008). Cellular and molecular mechanisms of fibrosis. J Pathol.

[11] Wynn TA, Vannella KM (2016). Macrophages in Tissue Repair, Regeneration, and Fibrosis. Immunity.

[12] Bringardner BD, Baran CP, Eubank TD, Marsh CB (2008). The role of inflammation in the pathogenesis of idiopathic pulmonary fibrosis. Antioxid Redox Signal.

[13] Reynolds HY (2005). Lung inflammation and fibrosis: an alveolar macrophage-centered perspective from the 1970s to 1980s. Am J Respir Crit Care Med.

[14] Fujii T (2002). Interaction of alveolar macrophages and airway epithelial cells following exposure to particulate matter produces mediators that stimulate the bone marrow. Am J Respir Cell Mol Biol.

[15] Tao F, Kobzik L (2002). Lung macrophage-epithelial cell interactions amplify particle-mediated cytokine release. Am J Respir Cell Mol Biol.

[16] Manzer R, Dinarello CA, McConville G, Mason RJ (2008). Ozone exposure of macrophages induces an alveolar epithelial chemokine response through IL-1alpha. Am J Respir Cell Mol Biol.

[17] Young LR (2016). Epithelial-macrophage interactions determine pulmonary fibrosis susceptibility in Hermansky-Pudlak syndrome. JCI Insight.

[18] Araki T, Milbrandt J (1996). Ninjurin, a novel adhesion molecule, is induced by nerve injury and promotes axonal growth. Neuron.

[19] Araki T, Zimonjic DB, Popescu NC, Milbrandt J (1997). Mechanism of homophilic binding mediated by ninjurin, a novel widely expressed adhesion molecule. J Biol Chem.

[20] Ahn BJ (2014). Ninjurin1 enhances the basal motility and transendothelial migration of immune cells by inducing protrusive membrane dynamics. J Biol Chem.

[21] Ifergan I (2011). Role of Ninjurin-1 in the migration of myeloid cells to central nervous system inflammatory lesions. Ann Neurol.

[22] Lee HJ, Ahn BJ, Shin MW, Choi JH, Kim KW (2010). Ninjurin1: a potential adhesion molecule and its role in inflammation and tissue remodeling. Mol Cells.

[23] Chen JS (2001). Identification of novel markers for monitoring minimal residual disease in acute lymphoblastic leukemia. Blood.

[24] Ahn BJ (2009). Ninjurin1 is expressed in myeloid cells and mediates endothelium adhesion in the brains of EAE rats. Biochem Biophys Res Commun.

[25] Tajouri L, Fernandez F, Griffiths LR (2007). Gene expression studies in multiple sclerosis. Curr Genomics.

[26] Moeller A, Ask K, Warburton D, Gauldie J, Kolb M (2008). The bleomycin animal model: a useful tool to investigate treatment options for idiopathic pulmonary fibrosis?. Int J Biochem Cell Biol.

[27] Jennewein C (2015). Contribution of Ninjurin1 to Toll-like receptor 4 signaling and systemic inflammation. Am J Respir Cell Mol Biol.

[28] Wilson MS, Wynn TA (2009). Pulmonary fibrosis: pathogenesis, etiology and regulation. Mucosal Immunol.

[29] Wynn TA, Ramalingam TR (2012). Mechanisms of fibrosis: therapeutic translation for fibrotic disease. Nat Med.

[30] Lebrun A (2017). CCR2+ monocytic myeloid-derived suppressor cells (M-MDSCs) inhibit collagen degradation and promote lung fibrosis by producing transforming growth factor-β1. J Pathol.

[31] Biernacka A, Dobaczewski M, Frangogiannis NG (2011). TGF-β signaling in fibrosis. Growth Factors.

[32] Zoz DF, Lawson WE, Blackwell TS (2011). Idiopathic pulmonary fibrosis: a disorder of epithelial cell dysfunction. Am J Med Sci.

[33] de Boer WI (2000). Monocyte chemoattractant protein 1, interleukin 8, and chronic airways inflammation in COPD. J Pathol.

[34] Manicone AM (2009). Role of the pulmonary epithelium and inflammatory signals in acute lung injury. Expert Rev Clin Immunol.

[35] Ahn BJ (2014). Ninjurin1 deficiency attenuates susceptibility of experimental autoimmune encephalomyelitis in mice. J Biol Chem.

[36] Wynn TA (2011). Integrating mechanisms of pulmonary fibrosis. J Exp Med.

[37] Wynn TA, Barron L (2010). Macrophages: master regulators of inflammation and fibrosis. Semin Liver Dis.

[38] Lee HK, Lee H, Luo L, Lee JK (2016). Induction of Nerve Injury-Induced Protein 1 (Ninjurin 1) in Myeloid Cells in Rat Brain after Transient Focal Cerebral Ischemia. Exp Neurobiol.

[39] Yang J (2013). Activated alveolar epithelial cells initiate fibrosis through secretion of mesenchymal proteins. Am J Pathol.

[40] Artlett CM (2012). The Role of the NLRP3 Inflammasome in Fibrosis. Open Rheumatol J.

[41] Byrne AJ, Maher TM, Lloyd CM (2016). Pulmonary Macrophages: A New Therapeutic Pathway in Fibrosing Lung Disease?. Trends Mol Med.

[42] Yin GN (2014). Inhibition of Ninjurin 1 restores erectile function through dual angiogenic and neurotrophic effects in the diabetic mouse. Proc Natl Acad Sci USA.

[43] Jiang D (2010). Inhibition of pulmonary fibrosis in mice by CXCL10 requires glycosaminoglycan binding and syndecan-4. J Clin Invest.

[44] Leng D (2013). Meta-analysis of genetic programs between idiopathic pulmonary fibrosis and sarcoidosis. PLoS One.

[45] Zhang, X., Goncalves, R. & Mosser, D. M. The isolation and characterization of murine macrophages. Curr Protoc Immunol Chapter 14, Unit14.11, 10.1002/0471142735.im1401s83 (2008). 10.1002/0471142735.im1401s83 PMC2834554 19016445

[46] Woo JK (2017). Lectin, Galactoside-Binding Soluble 3 Binding Protein Promotes 17-N-Allylamino-17-demethoxygeldanamycin Resistance through PI3K/Akt Pathway in Lung Cancer Cell Line. Mol Cancer Ther.

[47] Jang YS (2016). Ninjurin1 suppresses metastatic property of lung cancer cells through inhibition of interleukin 6 signaling pathway. Int J Cancer.

[48] Hübner RH (2008). Standardized quantification of pulmonary fibrosis in histological samples. Biotechniques.

[49] Livak KJ, Schmittgen TD (2001). Analysis of relative gene expression data using real-time quantitative PCR and the 2(−Delta Delta C(T)) Method. Methods.

