# Supplementary material for: Ninjurin1 Plays a Crucial Role in Pulmonary Fibrosis by Promoting Interaction between Macrophages and Alveolar Epithelial Cells
Source: Sci Rep. 2018 Dec 3;8:17542. doi: 10.1038/s41598-018-35997-x (PMC6277454; doi:10.1038/s41598-018-35997-x)
Supplement: Supplementary file 1 — Supplementary Information [file 41598_2018_35997_MOESM1_ESM.docx]

**Ninjurin1 Plays a Crucial Role in Pulmonary Fibrosis by Promoting Interaction between Macrophages and Alveolar Epithelial Cells**

**Seungho Choi^1*^, Jong Kyu Woo^2*^, Yeong-Su Jang^3^, Ju-Hee Kang^3^, Jong-Ik Hwang^4^, Je-Kyung Seong^2^, Yeo Sung Yoon^1#^, Seung Hyun Oh^3#^**

^1^College of Veterinary Medicine, Seoul National University, Seoul, Republic of Korea

^2^Korea Mouse Phenotyping Center, College of Veterinary Medicine, Seoul National University, Seoul, Republic of Korea

^3^College of Pharmacy, Gachon University, Incheon, Republic of Korea

^4^Graduate School of Medicine, Korea University, Seoul, Republic of Korea

**Corresponding authors**

**Seung Hyun Oh**

College of Pharmacy, Gachon University, Incheon, Republic of Korea

E-mail: eyeball@hanmail.net; Phone: + 82-32-820-4929; Fax: +82-32-820-4829

**Yeo Sung Yoon**

College of Veterinary Medicine, Seoul National University, Seoul, Republic of Korea

E-mail: ysyoon@snu.ac.kr; Phone: + 82-2-880-1264

**^#, *^** These authors contributed equally to this study

**Supplementary Materials and Methods**

**Antibodies**

The primary antibodies used for western blotting were anti-p-p65 (Cell Signaling Technology, MA, USA, cat. #. 3033), anti-p65, (Cell Signaling Technology, cat. #. 8242), anti-α-tubulin (Sigma, MO, USA, cat. #. cp06), anti-p-SMAD3 (Cell Signaling Technology, cat. #. 9520), anti-GAPDH (Merck Millipore, Darmstadt, Germany, CB1001), and anti-Ninj1 (Abclone, Seoul, Korea, customized). All primary antibodies for western blotting were diluted to 1:1000 except for anti-Ninj1 antibody, 1:3000. The primary antibodies for immunohistochemistry were anti-Ninjurin1 (Abclone, 1:2000), anti-ER-TR7 (Santa Cruz, CA, USA, cat. #. sc-73355, 1:200), and anti-α-SMA (Sigma-Aldrich, cat. #. A2547, 1:1000). The primary antibodies for immunofluorescence assay were anti-SMAD2/3 (Cell Signaling Technology, #3102, 1:500), anti-Ninjurin1 (Abclone, 1:1000). anti-F4/80 FITC (eBioscience, CA, USA, cat. #. 11-4801, 1:500) and anti-mouse α-SMA (Sigma-Aldrich, 1:1000). For FACS analysis, anti-Ninjurin1 (Abclone, 1:200), Alexa Fluor 647 anti-CD45 antibody (BD Bioscience, CA, USA, 103124, 1:200), PE anti-mouse CD11b antibody (BD Bioscience, cat. #. 553311, 1:200), FITC rat anti-mouse CD3 antibody (BD Pharminogen, CA, USA, cat. #. 555274, 1:200) and FITC rat anti-mouse CD19 (BD Pharminogen, CA, USA, cat. #. 553785, 1:200) were applied.

**Primers for reverse transcription- and real-time PCR**

All primers were purchased from Cosmogenetech co, Ltd. (Seoul, Korea). The primers for reverse transcription-PCR are the followings: IL-1β, GCC TTG GGC CTC AAA GGA AAG AAT C (forward) and GGA AGA CAC AGA TTC CAT GGT GAA G (reverse); iNOS, GTG GTG ACA AGC ACA TTT GG (Forward) and GGC TGG ACT TTT CAC TCT GC (reverse); TGF-β1, CTT CAG CTC CAC AGA GAA GA (forward) and CAC GAT CAT GTT GGA CAA CTG (reverse); col1a1, GAC CTC AAG ATG TGC CAC TC (forward) and CAA GGG TGC TGT AGG TGA AG (reverse); CXCL1, GAG CTG CGC TGT CAG TGC CT (forward) and TGC CAT CAG AGC AGT CTG TC (reverse); α-SMA, GAA GAG CTA CGA ACT GCC TG (forward) and CAG ACA GAG TAC TTG CGT TC (reverse); TNFα, ATA GCT CCC AGA AAA GCA AGC (forward) and CAC CCC GAA GTT CAG TAG ACA (reverse); MUC5B, CAG CAA ACG TCG TCA ACT GG (forward) and TTG GGT TGG CAG AGT GTT GT (reverse); β-actin: TGG AAT CCT GTG GCA TCC ATG AAA C (forward) and TAA AAC GCA GCT CAG TAA CAG TCC G (reverse).

**Primers for recombinant mouse Ninj1^1-50^**

Recombinant protein was generated as described in our previous report^1^. In order to produce 1-50 a.a. of mouse Ninj1, Ninj1-encoding gene was amplified from mouse cDNA using the following primers: Ninj1^1-50^, GCC CAA GCT TCC ATG GAG TCG GGC (forward) and CT AGC TCG AGT GCT CTC CGC AGC GCT (reverse).

**Preparation of bronchoalveolar lavage fluid (BALF) and BAL cells**

Before excision of lungs, cell-free bronchoalveolar lavage fluid (BALF) and BAL cells were collected as described in the previous report^2^. After each mouse was euthanized by CO_2_, the lung and trachea were surgically exposed. Six hundred μl of PBS was injected and aspirated through trachea. This step was proceeded total 3 times. Total volume of BALF collected was about 1.5 ml. BALF was centrifuged at 1500 rpm for 3 minutes. The cell pellet was resuspended in 1 ml of PBS and subjected to inflammatory cell analysis. The supernatant, which is cell-free BALF, was stored at -20 °C. BALF was subjected to ELISA to evaluate the amount of IL-1β and TGF-β1. Cell-free BALF was also used to stimulate primary fibroblast.

**Primary cell isolation**

Lung primary fibroblasts were isolated from lungs of C57BL/6J mice. The whole lungs were aseptically excised and placed on petri dish. The lungs were minced until the size of lung fragments is less than 2 mm. The lung fragments were placed in tissue culture dish in DMEM supplemented with 15% FBS and streptomycin (100 µg/ml)/penicillin (100 units/ml) and incubated in a humidified CO_2_ incubator without changing media until crawling fibroblasts from tissue fragments were observed. The media was replaced with the fresh media and incubated until no tissue fragments was observed. The primary fibroblasts were subcultured and incubated in a humidified CO_2_ incubator for additional 7 days. The culture media was changed every 2~3 days.

Peritoneal macrophages were isolated from WT or Ninj1 KO mice as described in the previous report^3^. The abdominal skin of the mouse was removed to expose peritoneal wall. To collect peritoneal cells, 5 ml of PBS was injected through peritoneal wall, using 10-ml syringe with 20 G needle, and aspirated PBS from peritoneum. This step was repeated twice. Peritoneal cells were centrifuged for 10 min at 400 x g. The cells were resuspended in DMEM supplemented with 10% FBS and streptomycin (100 µg/ml)/penicillin (100 units/ml), and seeded on culture plates. The cells were incubated in a humidified CO_2_ incubator for 1 hour and washed with PBS 3 times in order to remove nonadherent cells. The adherent cells are over 90% macrophages.Peritoneal macrophages were isolated from WT or Ninj1 KO mice as described in the previous report^3^. The abdominal skin of the mouse was removed to expose peritoneal wall. To collect peritoneal cells, 5 ml of PBS was injected through peritoneal wall, using 10-ml syringe with 20 G needle, and aspirated PBS from peritoneum. This step was repeated twice. Peritoneal cells were centrifuged for 10 min at 400 x g. The cells were resuspended in DMEM supplemented with 10% FBS and streptomycin (100 µg/ml)/penicillin (100 units/ml), and seeded on culture plates. The cells were incubated in a humidified CO_2_ incubator for 1 hour and washed with PBS 3 times in order to remove nonadherent cells. The adherent cells are over 90% macrophages.

**Hydroxyproline assay**

The amount of collagens accumulated in the lungs or secreted from primary fibroblasts were measured using the Hydroxyproline Colorimetric Assay Kit (BioVision, CA, USA), following the manufacturer’s instruction. Briefly, the whole lung specimens were homogenized in 100 μl distilled water per 10 mg lung tissue. One hundred μl of hydrochloric acid (~12N) was added to 100 μl of each lung homogenate. For conditioned media, 100 µL of each sample was taken and 100 µL of HCl was added to each sample. The samples were hydrolyzed at 120℃ for 3 hours. Samples were vortexed and centrifuged at 10,000xg for 3 min. Fifty μl of each hydrolyzed sample was transferred to 96-well plate and dried in a 60 °C oven. For standard curve preparation, 0.1, 0.2, 0.4, 0.6, 0.8 and 1 µg of hydroxyproline standards were prepared in 96-well plate. One hundred μl of DMAB was added to each sample and incubated at 60 °C for 90 min, following incubation with 100 μl of Chloramine T reagent at room temperature for 5 min. The absorbance was measured at 560 nm in microplate reader. The amount of hydroxyproline was calculated and expressed in μg/mg lung tissue.

**Immunohistochemistry**

Immunohistochemistry (IHC) was performed according to the protocol in our previous report^4^. The 5 µm-thick sections of paraffin-embedded lung tissue were deparaffinized and rehydrated. The endogenous peroxidase was blocked with 3% H_2_O_2_ for 30 minutes at room temperature and washed 3 times with distilled water. Antigen retrieval was then performed by heating the sections in boiling 10 mM citrate supplemented with 0.1% Tween 20 and 0.5% EDTA in microwave oven for 6 minutes and washed the sections 3 times with PBS-T (0.05% Tween 20). In order to block endogenous mouse Ig, the sections were incubated in MOM^TM^ Mouse Ig Blocking Reagent (Vector Laboratories, CA, USA) for 1 hour at room temperature. The sections were then blocked by using Antibody Diluent Reagent Solution (Invitrogen, CA, USA) for 30 minutes. The sections were incubated with primary antibodies overnight at 4 °C and washed with PBS+0.1% Tween 20 (PBS-T) 3 times, followed by secondary antibody conjugation for 1 hour. The sections were washed with PBS-T 3 times and incubated with streptavidin-HRP solution for 15 minutes at room temperature. After washing with PBS-T 3 times, detection was performed using 3,3-diaminobenzidine (DAB) substrate solution (Dako, Carpinteria, CA, USA). The sections were washed in distilled water, counterstained with hematoxylene, dehydrated and mounted. The primary antibodies for immunohistochemistry were anti-Ninjurin1 (Abclone, 1:2000), anti-ER-TR7 (Santa Cruz, CA, USA, sc-73355, 1:200), and anti-α-SMA (Sigma-Aldrich, A2547, 1:1000).

**Immunofluorescence assay**

Immunofluorescence assay was performed according to the protocol in our previous report^4^. The 5 µm-thick sections of paraffin-embedded lung tissue were deparaffinized and rehydrated. Antigen retrieval was then performed by heating the sections in boiling 10 mM citrate supplemented with 0.1% Tween 20 and 0.5% EDTA in microwave oven for 6 minutes and washed the sections 3 times with PBS-T (0.05% Tween 20). For cells, they were cultured on the cover slides in 12-well plates. The cells were fixed in an acetone-methanol (1:1) mixture for 30 minutes at -20 °C. The tissue sections and cells were blocked with Antibody Diluent Reagent Solution (Invitrogen) for 1 hour. The samples were incubated with primary antibodies overnight at 4 °C and secondary antibodies for 2 hours at room temperature. After mounting the slides with VectaShield Mounting Medium with DAPI (Vector Laboratories), the slides were sealed with nail polish and stored at -20 °C until observation using a confocal microscope (Nikon Instruments Inc., New York, USA). The primary antibodies are anti-SMAD3 (Abcam, OR, USA, ab28379, 1:500), anti-F4/80 FITC (eBioscience, CA, USA, 11-4801, 1:500) and anti-mouse α-SMA (Sigma-Aldrich, 1:1000).

**Semiquantitative real-time PCR**

Semiquantitative real-time PCR (qPCR) was performed using Stratagene Mx3000P QPCR system (Agilent Technologies, CA, USA). PCR conditions were one cycle of 10 min at 95 °C, 40 cycles of 30 sec at 95 °C, 30 sec at 60 °C and 30 sec at 72 °C. The mRNA expression of each sample was calculated by 2^-ΔΔCt^ method^5^. Each sample was analyzed in triplicate.

**Western blot analysis**

Western blot was performed as described in our previous report^4^. Briefly, cells or tissue specimens were lysed using lysis buffer containing 20 mM Tris – HCl (pH 7.6), 1 mM EDTA, 140 mM NaCl, 1% Nonidet P-40, 1 mM sodium fluoride, and 1 mM sodium vanadate. The concentration of protein was determined by using a bicinchoninic acid protein assay kit (Pierce, IL, USA). Protein samples were prepared by supplementing with sodium dodecyl sulfate (SDS)-sample buffer and boiling for 5 min at 95 °C. SDS-polyacrylamide gel electrophoresis (PAGE) was then performed to separate the protein by size. The proteins were transferred to polyvinyldene fluoride membrane (PALL Life Science, NY, USA). The proteins on the membrane was blocked in 5% skim milk and washed with TBS-T (0.01% Tween 20). The primary antibodies were conjugated overnight at 4 and the secondary antibodies were conjugated for 1 hour at room temperature. Detection was performed by using Absignal (Abclone, Seoul, Korea) according to the manufacturer’s instruction. All western blot results were semi-quantified by using Image J program.

**Supplementary Figures**

**Supplementary Fig. S1.**


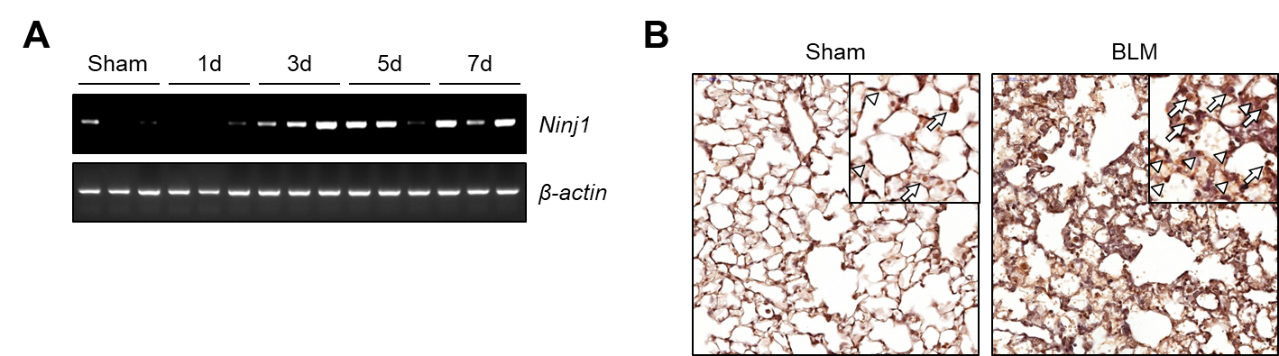


**Supplementary Fig. S2.**

**
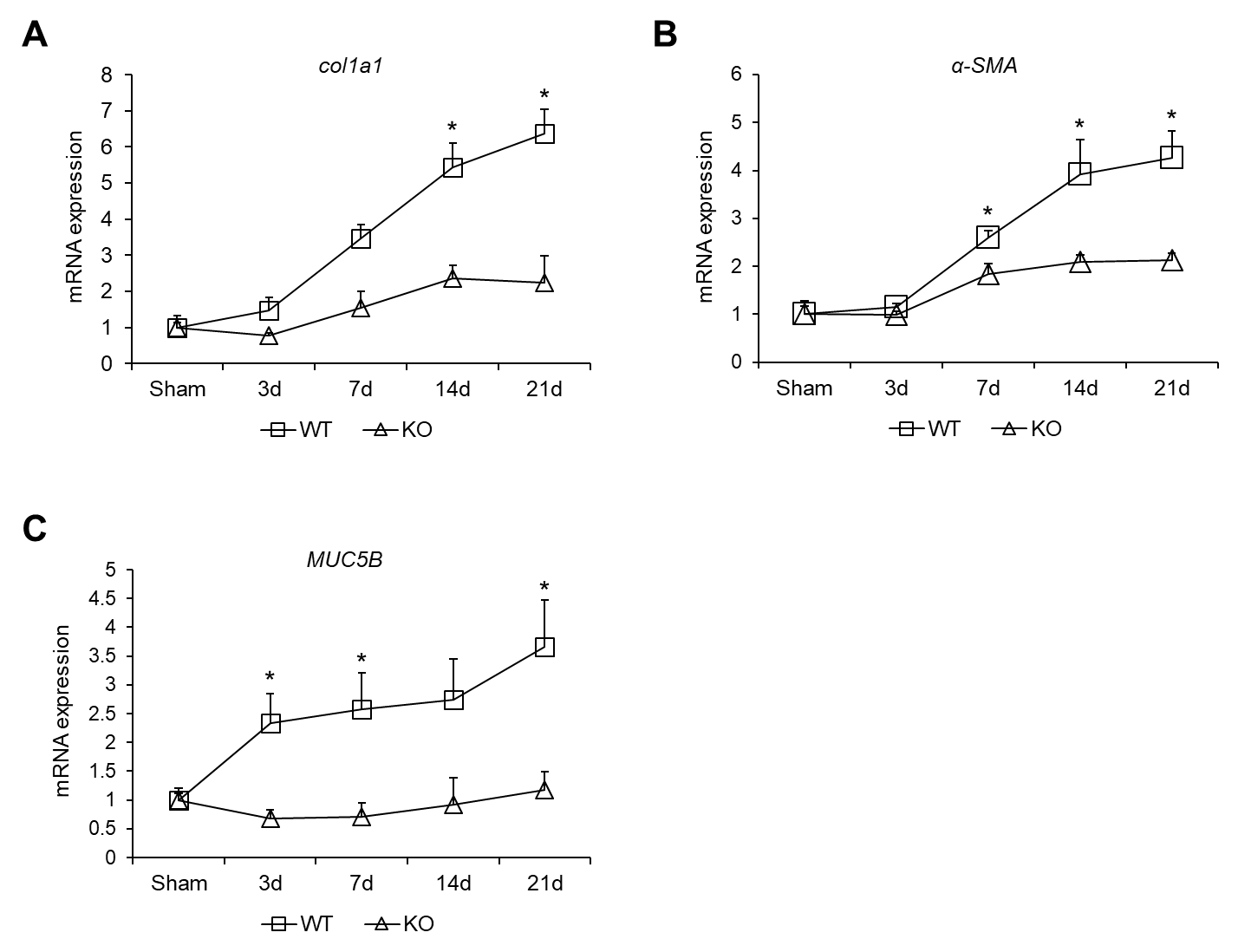
**

**Supplementary Fig. S3.**

**
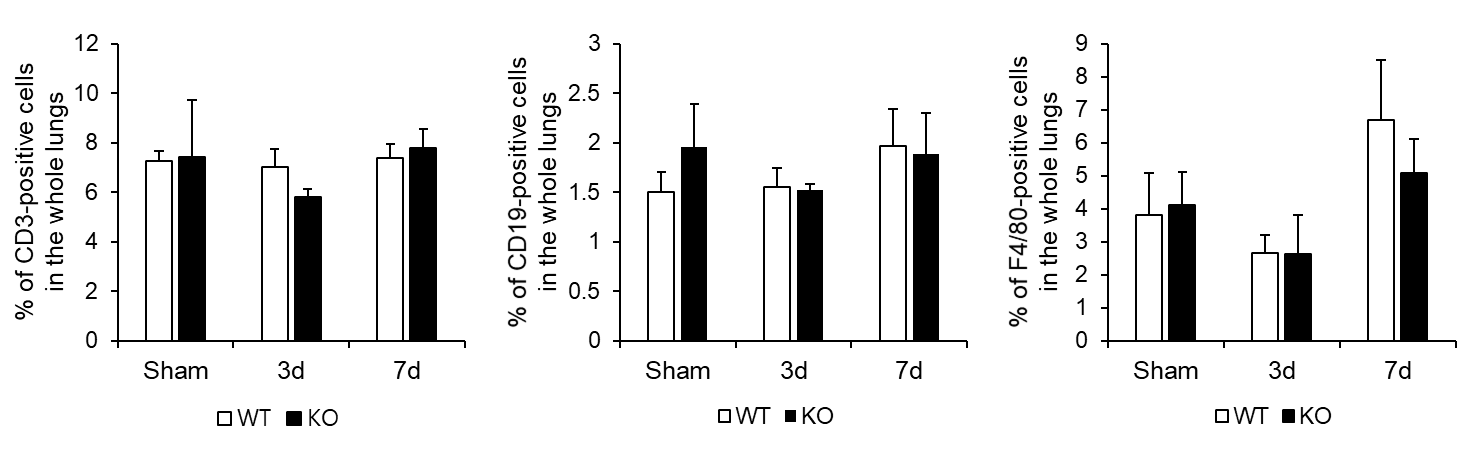
**

**Supplementary Fig. S4.**

**
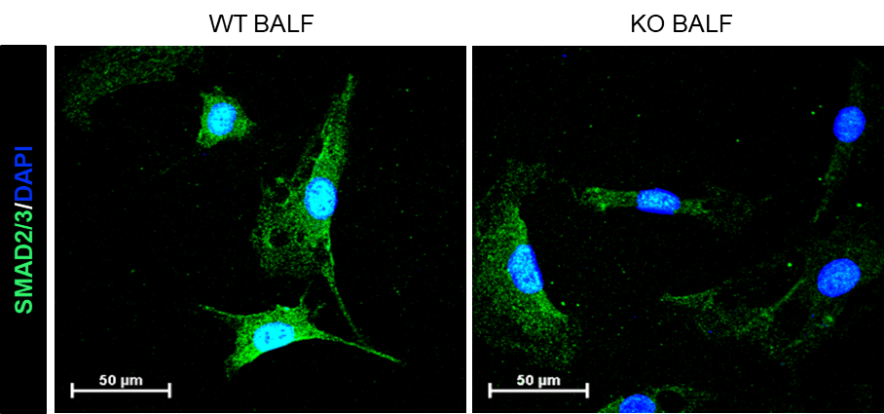
**

**Supplementary Fig. S5.**

**
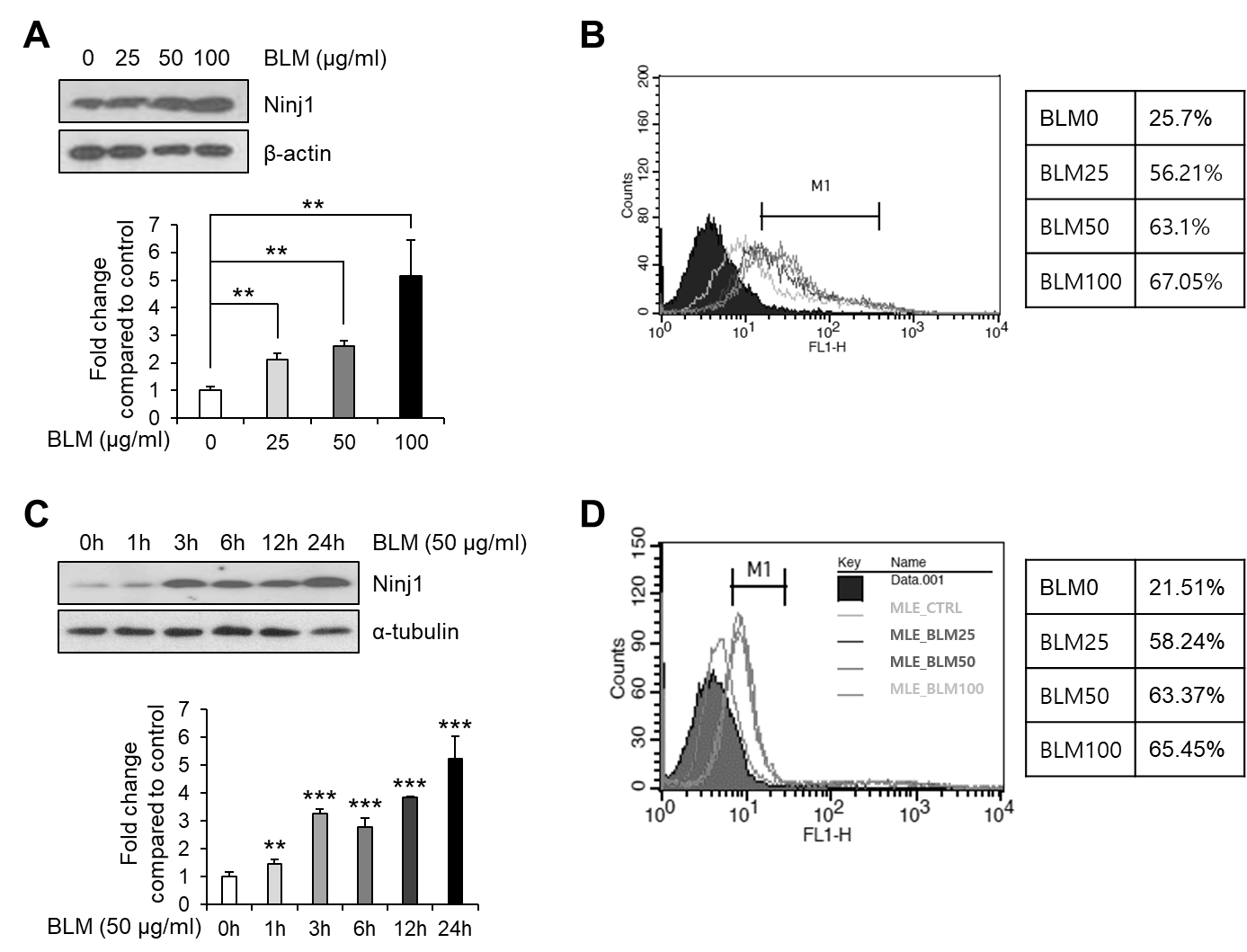
**

**Supplementary Fig. S6.**

**
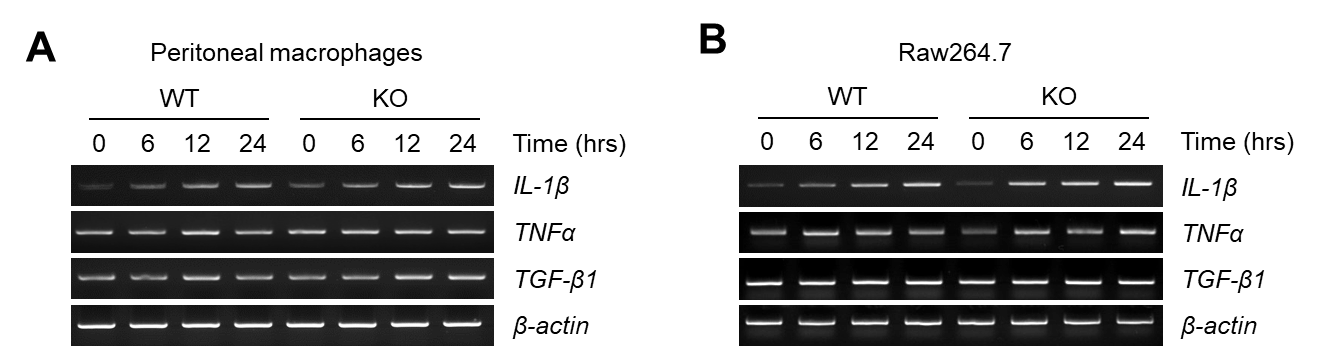
**

**Supplementary Fig. S7.**

**
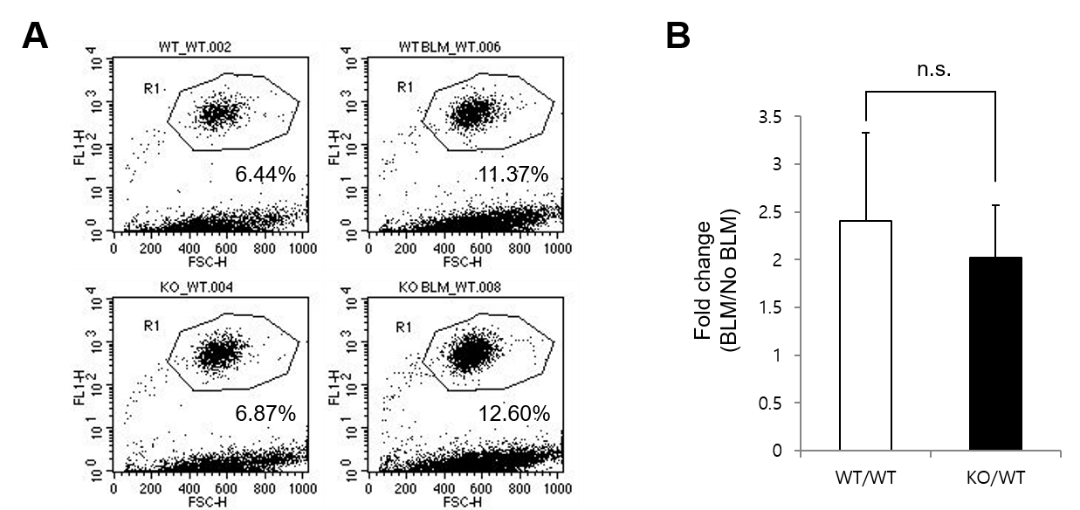
**

**Supplementary Fig. S8.**


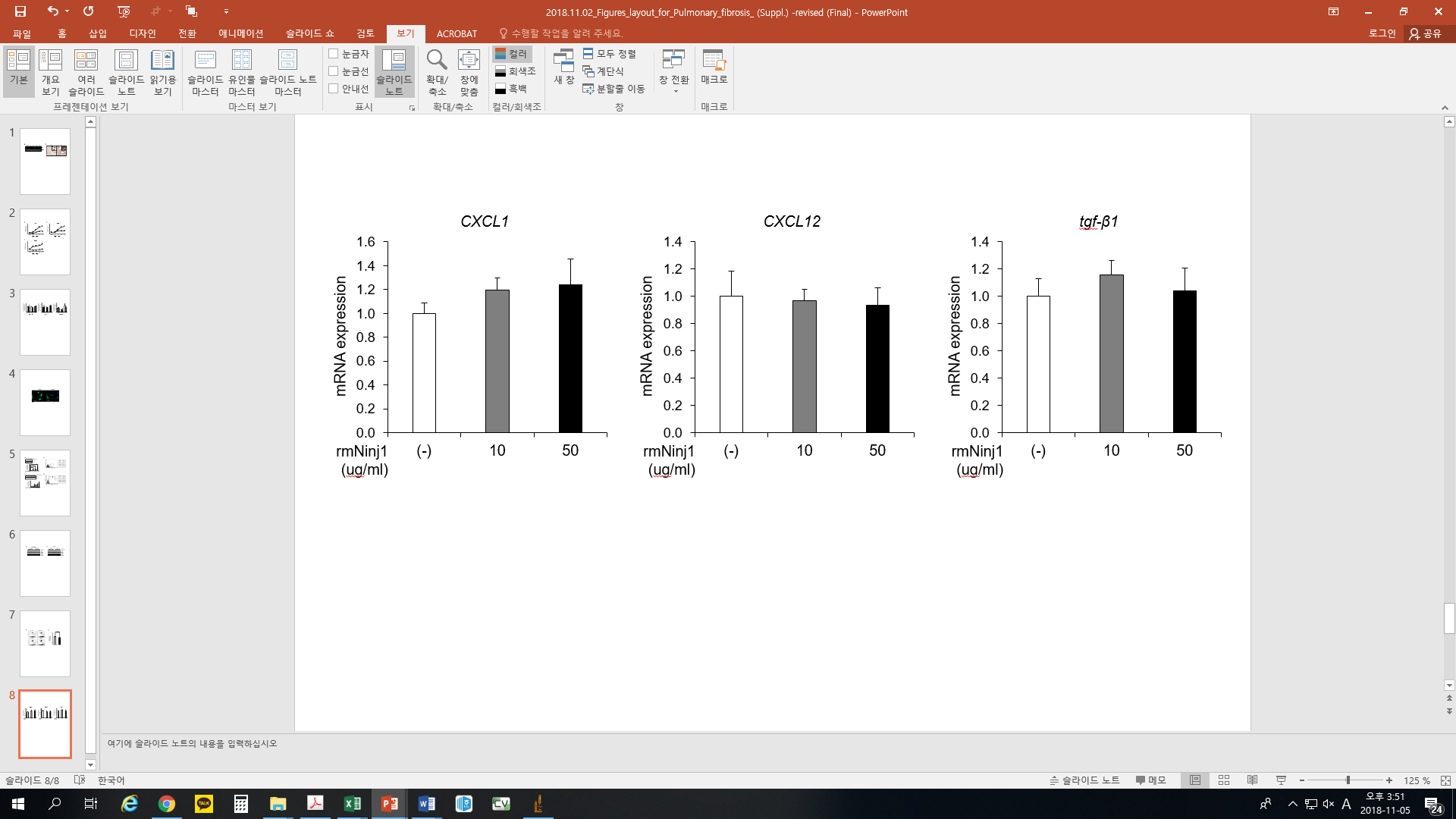


**Supplementary Figure Legends**

**Supplementary Fig. S1.**

The expression of Ninj1 is elevated in the lings of BLM-treated mice. (A) RT-PCR for *Ninj1* expression in the lungs of BLM-treated C57BL/6J mice in a time-dependent manner (n=3). (B) Representative images of immunohistochemistry for Ninj1 expression in the lungs of BLM-treated C57BL/6J mice at day 7 (n=3; arrows, macrophages; arrow heads, AECs).

**Supplementary Fig. S2.**

Pulmonary fibrosis was attenuated in BLM-treated Ninj1 KO mice. Semi-quantitative real-time PCR for mRNA expression of *col1a1* (A), *α-SMA* (B) and *MUC5B* (C) in the lungs of BLM-treated WT or Ninj1 KO mice in a time-dependent manner (n=3). The semi-quantitative real-time PCR data are expressed in means ± SEM of triplicates. **p*<0.05.

**Supplementary Fig. S3.**

Inflammatory cell population is not different between BLM-treated WT and Ninj1 KO mice. Inflammatory cell population was analyzed by FACS analysis using the digested whole lung cells from each mouse (n=3). CD45^+^CD3^+^, CD45^+^CD19^+^ and CD45^+^F4/80^+^ cells were analyzed. The data are expressed in means ± SD of triplicates.

**Supplementary Fig. S4.**

Activation of primary fibroblast is diminished when treated with BALF from BLM-treated Ninj1 KO mice. Representative images of immunofluorescence assay to determine nuclear localization of SMAD3. The results are representative of three independent experiments.

**Supplementary Fig. S5.**

The expression of Ninj1 is elevated by inflammatory stimuli in Raw264.7 and MLE-12. (A) Western blot analysis for Ninj1 expression in Raw264.7 cells with or without BLM treatment. Representative images (upper) and quantification (lower) of western blot. (B) Representative images (left) and numerical values (right) of flow cytometry analysis for Ninj1 expression in Raw264.7 cells with or without BLM treatment. (C) Western blot analysis for Ninj1 expression in MLE-12 with or without BLM treatment. Representative images (upper) and quantification (lower) of western blot. (D) Representative image (left) and numerical values (right) of flow cytometry analysis for Ninj1 expression in MLE-12 with or without BLM treatment. The results are representative of three independent experiments. The data are expressed in means ± SD of triplicates. ***p*<0.01; ****p*<0.001.

**Supplementary Fig. S6.**

Ninj1 deficiency does not alter the production of inflammatory cytokines. RT-PCR to assess the expression of cytokines, *IL-1β*, *TNFα* and *TGF-β1*, in BLM (50 μg/ml)-treated WT and Ninj1 KO peritoneal macrophages (A) or Raw264.7 (B) in a time-dependent manner. The results are representative of three independent experiments.

**Supplementary Fig. S7.**

Deficiency of Ninj1 does not affect adhesion between Raw264.7 and MLE-12. (A) Representative images of flow cytometry analysis to assess the number of WT Raw264.7 cells bound to BLM-treated or untreated WT or Ninj1 KO MLE-12 cells. (B) Fold changes of the number of WT Raw264.7 cells bound to BLM-treated WT or Ninj1 KO MLE-12 cells, over the number of WT Raw264.7 cells bound to untreated WT or Ninj1 KO MLE-12 cells. The results are representative of three independent experiments and all numerical values are expressed in means ± SD of triplicates. ns = not significant.

**Supplementary Fig. S8.**

Recombinant Ninj1^1-50^ (rmNinj1) does not affect the expression of pro-inflammatory and pro-fibrotic cytokines in AECs. Semi-quantitative real-time PCR to assess the expression of the cytokines, *CXCL1*, *CXCL12* and *TGF-β1* in MLE-12 cells treated with rmNinj1^1-50^ (50 μg/ml). The results are representative of three independent experiments. The data are expressed in means ± SEM.

**References**

1 Woo, J. K. *et al.* Lectin, Galactoside-Binding Soluble 3 Binding Protein Promotes 17-N-Allylamino-17-demethoxygeldanamycin Resistance through PI3K/Akt Pathway in Lung Cancer Cell Line. *Mol Cancer Ther* **16**, 1355-1365, doi:10.1158/1535-7163.MCT-16-0574 (2017).

2 Jiang, D. *et al.* Inhibition of pulmonary fibrosis in mice by CXCL10 requires glycosaminoglycan binding and syndecan-4. *J Clin Invest* **120**, 2049-2057, doi:10.1172/JCI38644 (2010).

3 Zhang, X., Goncalves, R. & Mosser, D. M. The isolation and characterization of murine macrophages. *Curr Protoc Immunol* **Chapter 14**, Unit 14.11, doi:10.1002/0471142735.im1401s83 (2008).

4 Jang, Y. S. *et al.* Ninjurin1 suppresses metastatic property of lung cancer cells through inhibition of interleukin 6 signaling pathway. *Int J Cancer* **139**, 383-395, doi:10.1002/ijc.30021 (2016).

5 Livak, K. J. & Schmittgen, T. D. Analysis of relative gene expression data using real-time quantitative PCR and the 2(-Delta Delta C(T)) Method. *Methods* **25**, 402-408, doi:10.1006/meth.2001.1262 (2001).
